# Modulation of pulsed electric field induced oxidative processes in protein solutions by pro- and antioxidants sensed by biochemiluminescence

**DOI:** 10.1038/s41598-024-71626-6

**Published:** 2024-09-30

**Authors:** Kateřina Červinková, Petra Vahalová, Michaela Poplová, Tomáš Zakar, Daniel Havelka, Martin Paidar, Viliam Kolivoška, Michal Cifra

**Affiliations:** 1 Institute of Photonics and Electronics of the Czech Academy of Sciences, 18200 Prague, Czechia; 2 Department of Inorganic Technology, Faculty of Chemical Technology, University of Chemistry and Technology, Technická 5, 160 28 Prague, Czechia; 3 J. Heyrovský Institute of Physical Chemistry of the Czech Academy of Sciences, 18200 Prague, Czechia

**Keywords:** Biochemistry, Electrochemistry, Photochemistry, Physical chemistry, Biomedical engineering, Proteins, Biological physics, Biophysics, Biotechnology

## Abstract

Technologies based on pulsed electric field (PEF) are increasingly pervasive in medical and industrial applications. However, the detailed understanding of how PEF acts on biosamples including proteins at the molecular level is missing. There are indications that PEF might act on biomolecules via electrogenerated reactive oxygen species (ROS). However, it is unclear how this action is modulated by the pro- and antioxidants, which are naturally present components of biosamples. This knowledge gap is often due to insufficient sensitivity of the conventionally utilized detection assays. To overcome this limitation, here we employed an endogenous (bio)chemiluminescence sensing platform, which enables sensitive detection of PEF-generated ROS and oxidative processes in proteins, to inspect effects of pro-and antioxidants. Taking bovine serum albumin (BSA) as a model protein, we found that the chemiluminescence signal arising from its solution is greatly enhanced in the presence of $\hbox {H}_2 \hbox {O}_2$ as a prooxidant, especially during PEF treatment. In contrast, the chemiluminescence signal decreases in the presence of antioxidant enzymes (catalase, superoxide dismutase), indicating the involvement of both $\hbox {H}_2 \hbox {O}_2$ and electrogenerated superoxide anion in oxidation-reporting chemiluminescence signal before, during, and after PEF treatment. We also performed additional biochemical and biophysical assays, which confirmed that BSA underwent structural changes after $\hbox {H}_2\hbox {O}_2$ treatment, with PEF having only a minor effect. We proposed a scheme describing the reactions leading from interfacial charge transfer at the anode by which ROS are generated to the actual photon emission. Results of our work help to elucidate the mechanisms of action of PEF on proteins via electrogenerated reactive oxygen species and open up new avenues for the application of PEF technology. The developed chemiluminescence technique enables label-free, in-situ and non-destructive sensing of interactions between ROS and proteins. The technique may be applied to study oxidative damage of other classes of biomolecules such as lipids, nucleic acids or carbohydrates.

## Introduction

Intense electric field delivered in the form of short pulses, termed as a pulsed electric field (PEF), is increasingly applied in diverse research and industrial domains. The main applications are in clinical and experimental medicine enabling non-thermal tissue ablation^1^, electrochemotherapy^2–4^, and innovative drug and gene delivery methods^5–8^. Another large application area is in the food industry, where PEF facilitates non-thermal pasteurization^9,10^ and enhances yields of active food compounds extraction^11–13^. The rising interest is also in bio/nanotechnology, where PEF can be used to modulate biomolecular functions, such as protein complex self-assembly^14^ and enzyme activity^15^.

The fundamental physical mechanism of PEF involves manipulating biomolecular structures, such as membranes or proteins, through the action on mobile or bound electric charges, inducing functional changes in biomolecules. However, in parallel to these electrophysical effects, PEF also causes electrochemical effects^16,17^, such as dissolving of the material of the electrodes to the sample^18^ or the generation of reactive oxygen species (ROS)^19^, which are often underappreciated. In this paper, we demonstrate novel modulatory effects of pro- and antioxidants, which are commonly present in PEF-treated biomaterials, on the chemiluminescence^20,21^ in a protein sample, taking bovine serum albumin (BSA) as a model protein. These chemiluminescence measurements provide insights into oxidative processes occurring in biomolecular samples subjected to PEF treatment. ROS include a variety of oxidant molecules with very rich chemistry and biological functions ranging from signaling to cell damage^22^. Understanding mechanisms of ROS formation and their mutual interactions in simple inorganic systems is a prerequisite necessary for comprehending their impact on complex biological matter. Ultrasensitive chemiluminescence measurements represent an ideal toolkit for exploring the dynamics of ROS at electrified interfaces within PEF.

While PEF has conventionally targeted lipid membranes^23^, there is growing interest in understanding its effects on proteins, which constitute the majority of biomolecules in cells, and are essential food components, as well as potential bio-nanotechnological components. Computational molecular dynamics simulations^24,25^ have explored the impact of electric field on protein structure and dynamics^26^, predicting changes in shape, surface area^27^, dipole moment^28,29^, secondary structure^30,31^, and unfolding^32^. Experimental works have corroborated these findings, with changes observed in UV-circular dichroism spectra^33^, fluorescence of aromatic amino acid residues^34^, hydrophobic group analysis^35^, protein degradation^36^ and protein sulhydryl groups accessibility to molecules in solvent^37^ indicating structural alterations and unfolding.

Most of the samples and materials treated by PEF (cultures of cells in biological media, biological tissues, and food material) include free ions, making them electrically conductive. In such samples, PEF triggers a temporary migration of ions towards respective electrodes, where they undergo electrochemical (charge transfer) reactions. These may be followed by consecutive chemical reactions among electrogenerated species and sample components, leading to a broad spectrum of intermediates and products. These may include ROS^19^, which can interact with biomolecules in samples, potentially causing their oxidative damage. Although some studies have reported oxidative changes in lipids^38–40^ and cellular biosamples^41–47^ after PEF treatment, the oxidative effects of PEF on well-defined protein samples remain rather unexplored^48^. To address this knowledge gap, we employed here label-free, endogenous chemiluminescence-based experimental platform^49^ for sensing the oxidative effects of PEF-electrogenerated ROS on proteins, taking BSA as a model protein.

BSA is a versatile and extensively studied, water-soluble protein derived from bovine blood, commonly utilized in various biochemical and pharmaceutical applications. Composed of 583 amino acid residues and possessing a molecular weight of approximately 66.4 kDa, BSA is a relatively large globular protein, that consists of a single polypeptide chain organized into three homologous domains^50^. These structural features play a crucial role in its ability to transport and bind various species within biological systems such as fatty acids, hormones, bilirubin, and metal ions such as $\hbox {Zn}^{2+}$, $\hbox {Cu}^{2+}$, $\hbox {Ca}^{2+}$, $\hbox {Ni}^{2+}$, and $\hbox {Mg}^{2+}$, as well as metallic complexes^51–54^. BSA can also bind $\hbox {Fe}^{2+}$, but it is not its primary function and the interaction is relatively weak compared to specialized iron-binding proteins like transferrin^55^. Binding versatility not only facilitates the distribution and protection of these molecules in the bloodstream but BSA also plays a critical role in maintaining osmotic pressure, buffering pH, and serves as a reservoir for essential nutrients and ions^50^. The isoelectric point (pI) of BSA is in the range 4.7–5.0 ^56^, meaning that at physiological pH (7.4), the BSA molecule carries a negative net charge. The diffusion coefficient of BSA is $6.3~\times ~10^{-11}$ $\hbox {m}^2\, \hbox {s}^{-1}$ s (the value for $23~^{\circ }$C) ^57^, while its electrophoretic mobility is $1.4\times 10^{-8}$ $\hbox {m}^{2}\, \hbox {s}^{-1}\,\hbox {V}^{-1~}$^58^.

Oxidation of free amino acids and protein samples by ROS leads to chemiluminescence. Hydroxyl ($\hbox {HO}^{\cdot }$) radical is the most powerful ROS, attacking amino acid residues, leading to the formation of organic radicals. These radicals readily react with omnipresent triplet molecular oxygen $^3\hbox {O}_2$ to produce peroxyl radicals that undergo various further reactions, including dimerization and intramolecular cyclization, resulting in the formation of high-energy tetraoxides or dioxetanes, respectively. The decomposition of these intermediates leads to electronically excited species including triplet carbonyls $^3$(R$'$COR$''$)* and singlet oxygen $^1\hbox {O}_2$. These molecules return to their ground state while releasing excess energy as photons observed as chemiluminescence^59^. It has been observed that certain amino acids, such as phenylalanine, tryptophan, histidine, and cysteine, exhibit significantly higher levels of chemiluminescence when subjected to oxidative environment compared to others^60^. Among these, cysteine is the only amino acid that generates substantial chemiluminescence when oxidized in uncatalyzed reaction with $\hbox {H}_2\hbox {O}_2$^60^. The content of cysteine residues in BSA is four times higher than the average across the proteome (see Table SI 1-5)^61^, suggesting its significant contribution in the chemiluminescence signal.

Conventional methods for analyzing ROS and their effects on proteins often combine biophysical and biochemical approaches^21,62^. Biophysical techniques typically use spectroscopic methods, such as UV-visible absorption and emission (fluorescence) spectroscopy^63^, electron paramagnetic resonance spectroscopy^64^, electrochemical techniques^65,66^, or mass spectrometry-based techniques^67^. Biochemical techniques use chemical labels that react with ROS or products of ROS with proteins, which enables the quantification of oxidative damage. Examples include Brady’s analysis that determines the amount of carbonyl groups as protein oxidation markers^68^. Ellman’s method determines free (unreacted) sulfhydryl groups in the protein reflecting the extent of its oxidation^69^. Both Brady’s and Ellman’s methods were applied in this work to quantify the effects of ROS electrogenerated in PEF on BSA. Structural changes in proteins (exposure of hydrophobic regions, unfolding etc.) resulting from the oxidative damage can then be detected using the aforementioned biophysical techniques. In contrast to all these techniques, the endogenous chemiluminescence method we employ here offers a unique advantage because it does not require any external energy input to the sample or labeling of the sample.

We acknowledge that the biological materials treated by PEF in industry like food processing^70,71^ and medical applications like PEF ablation^1^, are chemically complex. Such materials (tissues, cells, cell extracts) naturally contain and generate prooxidants, such as hydrogen peroxide ($\hbox {H}_2\hbox {O}_2$)^72^ and antioxidants, such as catalase (CAT)^73^ and superoxide dismutase (SOD) antioxidant enzymes^74^, which were tested in the past in the context of PEF treatment of cell cultures^44^.

**Fig. 1:**
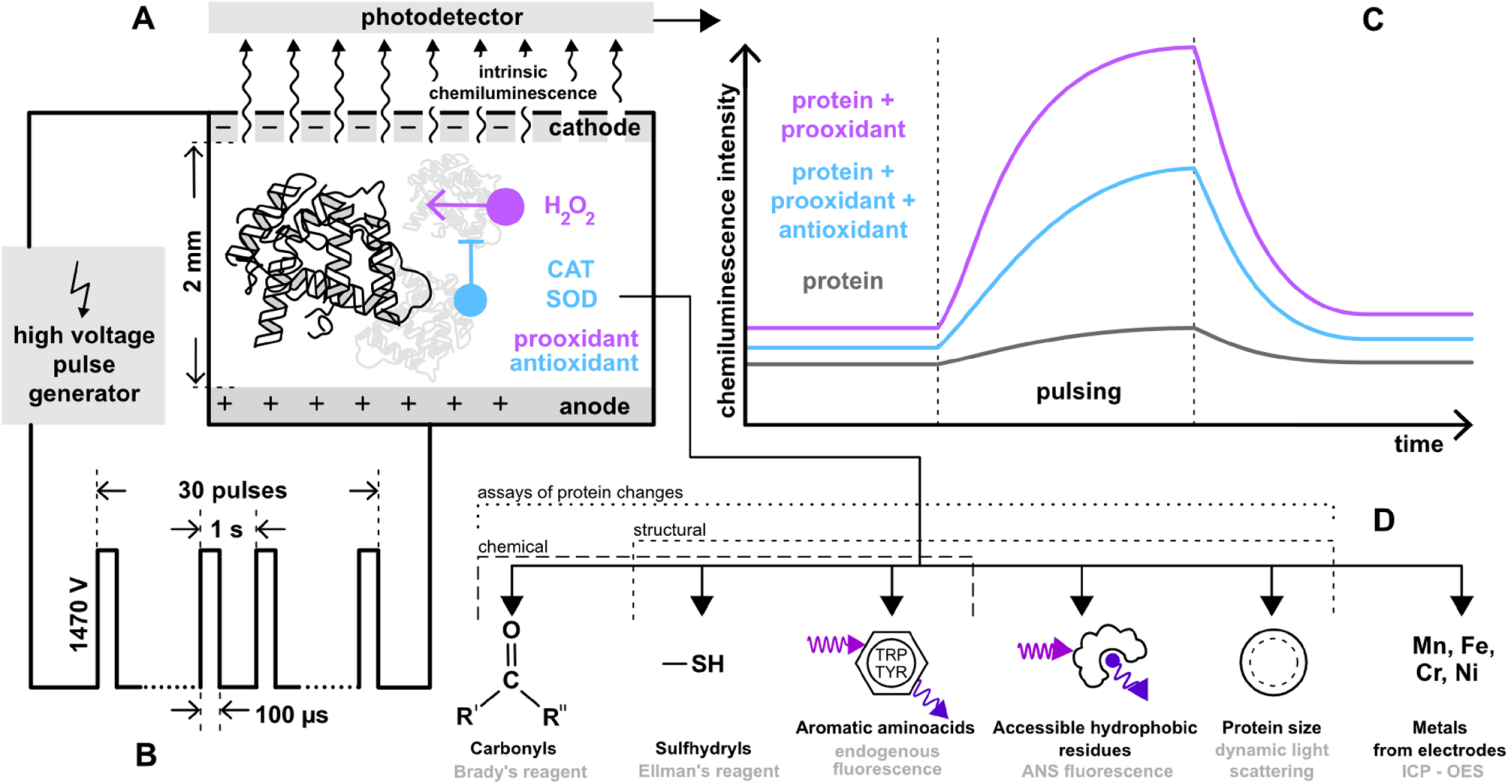
Overview of the main concepts and messages of the paper. The chemiluminescence signal (**C**) is generated from the solution of BSA (**A**) when exposed to a sequence of intense electric pulses (**B**). No external chemiluminescent labels are required; the signal arises due to components endogenous to the sample. The chemiluminescence reports on oxidative processes in the BSA solution. We analyzed the behavior of chemiluminescence in the presence or absence of prooxidant as well as enzymatic antioxidants and respective combinations. Furthermore, we used various biochemical and biophysical techniques (**D**) to analyze the effects of the PEF treatment on BSA.

Hence, it is crucial to comprehend how these substances modulate ROS-related reactions during PEF treatment. ROS electrogenerated in PEF in the absence and presence of prooxidant ($\hbox {H}_2\hbox {O}_2$) and interactions of ROS with BSA were detected by (bio)chemiluminescence measurements. High sensitivity was due to the preconcentration of BSA at anode by ionic migration in PEF. Further, we assessed chemical and structural changes in BSA using several techniques, such as carbonyl and sulfhydryl group determination, endogenous fluorescence from aromatic amino acid residues, and fluorescence probing of hydrophobic protein structures. We also propose a complex reaction scheme explaining how PEF-generated ROS induce protein oxidation leading to the biochemiluminescence and how this protein damage is modulated by $\hbox {H}_2\hbox {O}_2$ prooxidant and antioxidants enzymes (CAT and SOD). See Fig. 1 for the concept of this work.

## Results and discussion

### Design of experiments and overview of involved processes

**Fig. 2:**
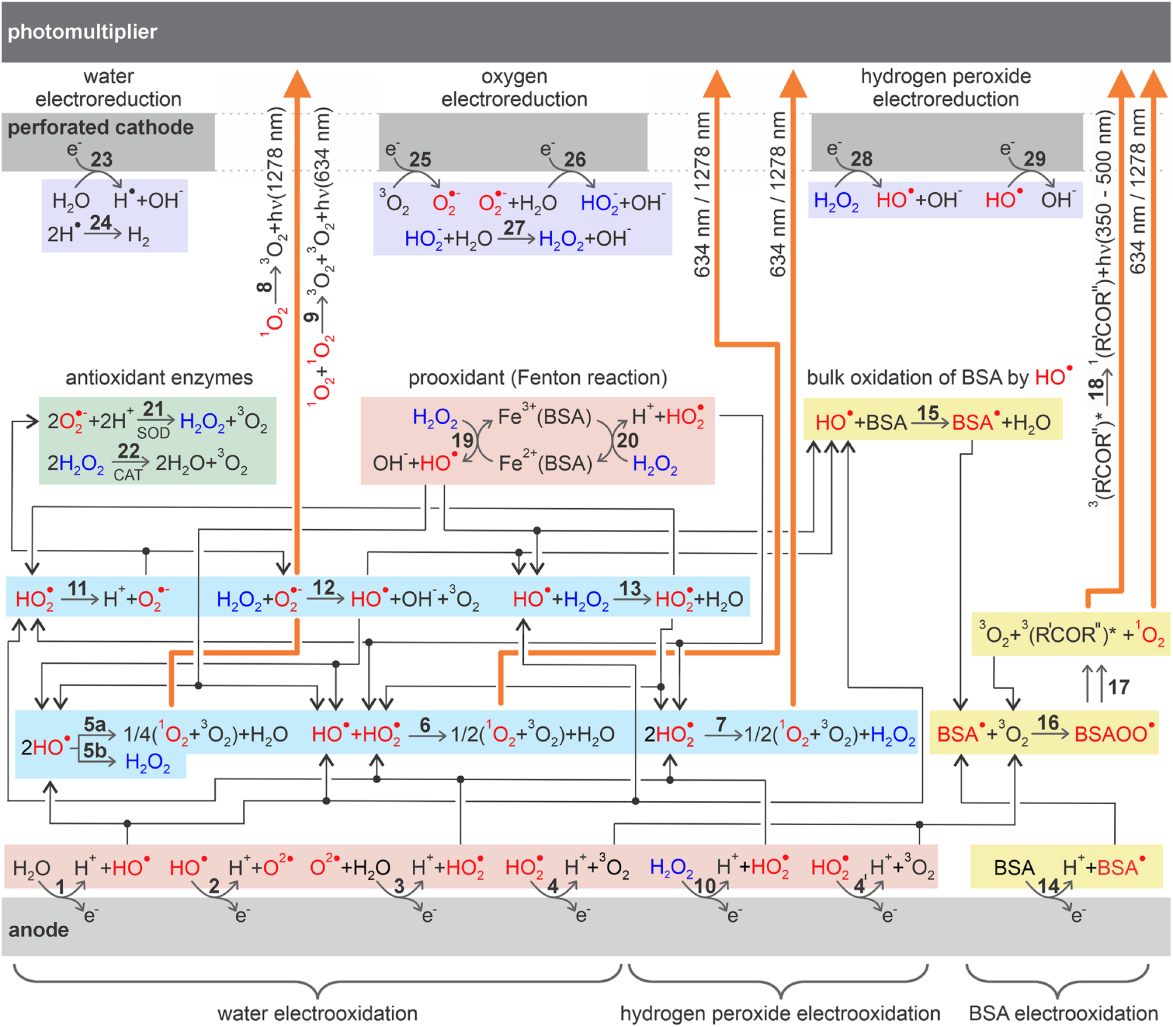
Overview of possible reactions in systems inspected in this work (PB and BSA) subjected to PEF, leading to the photon emission. Species shown as red denote ROS (radicals and $^1\hbox {O}_2$) and radical forms of BSA, blue denotes $\hbox {H}_2\hbox {O}_2$ (originally introduced in selected experiments) and its deprotonated form $\hbox {HO}_2^-$. Reactions are grouped to color fields based on their typology (anodic electrooxidation, bulk reactions among ROS, oxidation of BSA, enzymatic reactions, Fenton reaction, cathodic electroreduction).

To explore the effects of the PEF on proteins, we performed the PEF treatment on the aqueous solution of BSA (Fig. 1) with a concentration of 0.6 mM ($40~\textrm{mg}\, \hbox {mL}^{-1}$), corresponding to the natural concentration of albumin in the serum^75^. PEF treatment was performed in a chamber with the anode and cathode positioned in a parallel-plate thin-layer configuration, developed and presented in our previous work^49^. This geometry enabled the generation of spatially homogeneous electric field and hence straightforward theoretical prediction (see Supporting Information 2, SI 2) of quantities relevant to physical and (electro)chemical phenomena occurring in investigated systems (ionic migration, amounts and spatial distribution of electrogenerated ROS, etc.). The cathode was perforated, which enabled sensing of intermediates and products generated at the anode (where oxidative processes of our interest take place) by chemiluminescence measurements. In this work, both cathode and anode were made of steel, which is the most commonly used electrode material in the industrial use of PEF^76,77^. As a reference system, we took phosphate buffer (PB), as a purely inorganic medium, with identical electric conductivity ($0.033~\textrm{S}\, \hbox {m}^{-1}$) and pH (7.2 ± 0.2) as experimentally determined in this work for the 0.6 mM BSA solution. Both these characteristics were satisfied for PB with the composition of 0.612 mM $\hbox {NaH}_2\hbox {PO}_4$ and 1.411 mM $\hbox {Na}_2\hbox {HPO}_4$. Performing PEF in PB enabled chemiluminescence sensing of electrogenerated ROS in the absence of biomolecules at the same rate of charge transfer reactions and assessed thus the net contribution of the biochemiluminescence caused by processes involving BSA molecules. The electrochemical and bulk chemical reactions leading to photoemissive processes relevant to this work are summarized in the proposed reaction mechanism in Fig. 2. Rate constants of involved bulk chemical reactions are summarized in Table SI 1-1 in Supporting Information 1 (SI 1). To keep the complexity of the reaction scheme at an acceptable level, we decided not to include electrogeneration and further reactions of ozone $\hbox {O}_3$ as well as reactions of singlet oxygen $^1\hbox {O}_2$ with BSA as both these species are known to primarily attack aromatic structures in the molecules^78,79^, the damage of which was not observed in the current work (see below). Furthermore, rate constants of reactions of amino acids with singlet oxygen^80^ and ozone^79^ are several orders of magnitude lower than for reactions of amino acids with $\hbox {HO}^{\cdot }$ radical (see Table SI 1-1 for the list of rate constants). More complex mechanisms of reactions among ROS and their interactions with proteins and other biomolecules will be the subject of our forthcoming publications.

Figures 1 and 8A show the PEF profile applied in this work. It consisted of 30 rectangular voltage pulses (each with the duration of 100 $\upmu$s and magnitude of 1470 V, see Fig. 9 for exact voltage profile) delivered in 1 Hz frequency. For the employed chamber (cathode-to-anode gap of 2 mm, see Fig. 6 for the measurement circuit), this magnitude corresponds to the field strength of 0.735 MV $\hbox {m}^{-1}$ (Fig. 7), which is in the range of values used in typical PEF applications on proteins^34,81^. To evaluate baseline contributions, measurements of luminescence were commenced 60 s before switching on PEF (pre-pulsing period) and continued upon switching off the PEF (post-pulsing period) to evaluate remanent signals, see Fig. 10 for details. The current flowing through electrodes was monitored during the pulsing period. Figure 8B shows representative current transients obtained in the first pulse of PEF for PB and BSA samples. As expected for media with the same conductivity, the obtained current transients were almost identical, which holds true also for all other experimental combinations employed in this work (Fig. SI 1-4, SI 1-4). Small overshoots at the beginning and upon the termination of the pulse were attributed to contributions of charging and discharging of electric double layers formed at the electrode/electrolyte interfaces. The vast majority of the charge was due to the interfacial electron transfer reactions. The observed current (12.5 ± 0.8 A) corresponded well to the theoretical value (12.9 A) calculated for applied voltage, chamber geometry, and sample conductivity, see SI 2.

### Chemiluminescence and its interpretation

**Fig. 3:**
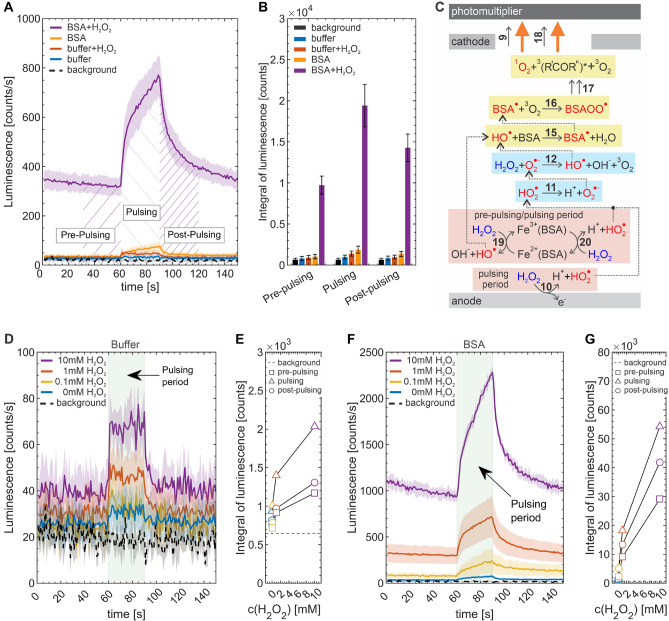
(**A**) Averaged luminescence transients obtained for PEF performed with PB and BSA solution, both in the absence and presence of $\hbox {H}_2\hbox {O}_2$ (1 mM). Hatched areas denote time intervals in which luminescence signal was integrated, with results shown in (**B**). (**C**) Summary of important processes leading to intensified chemiluminescence in the BSA/$\hbox {H}_2\hbox {O}_2$ system. Averaged luminescence transients obtained in PEF experiments with (**D**) PB and (**F**) BSA solution with varied $\hbox {H}_2\hbox {O}_2$ concentration. Individual luminescence signals were integrated in the pre-pulsing, pulsing, and post-pulsing period, with averaged results shown in (**E**) and (**G**). In (**A**), (**D**), (**F**) the standard deviation is in shaded error bars. (**E**, **G**) standard deviation values are in Table SI 1-2.

Figure 3A shows averaged luminescence transients obtained for PB and BSA samples, both in the absence and presence of 1 mM $\hbox {H}_2\hbox {O}_2$. The dashed black line depicts the averaged baseline signal recorded in the empty dry chamber. In the pre-pulsing period, signals obtained for PB, $\textrm{PB}/\hbox {H}_2\hbox {O}_2$, and BSA solution are almost identical to the baseline signal, implying no chemiluminescence. In the pulsing period, the signal recorded for PB was only slightly elevated, indicating a low concentration of species generating chemiluminescence. As was demonstrated in our recent contribution^49^, the magnitude of chemiluminescence observed at anode for PB dramatically depends on employed anode material, with steel having little response, in contrast to glassy carbon anode. Low signal on steel was explained by a fast conversion of ROS generated as reaction intermediates ($\hbox {HO}^{\cdot }$ and $\hbox {HO}_2^{\cdot }$, reactions 1 to 3 in Fig. 2) to non-emissive (ground state) triplet molecular oxygen $^3\hbox {O}_2$ (reaction 4) as the final product of anodic water electrooxidation^82^. We presume that small observed chemiluminescence is due to singlet oxygen $^1\hbox {O}_2$ formed by collisions among $\hbox {HO}^{\cdot }$ and $\hbox {HO}_2^{\cdot }$ (reactions 5a, 6 and 7)^83–86^. $^1\hbox {O}_2$ can emit light either by monomolecular decay (reaction 8, emission maximum at 1278 nm) or bimolecular decay (reaction 9, at 634 nm), both forming $^3\hbox {O}_2$^87^. The $^1\hbox {O}_2$ chemiluminescence observed in this work was only due to the bimolecular decay (the employed photomultiplier had practically zero quantum efficiency in the near IR region). Additionally to the disproportionation (reaction 5a), two $\hbox {HO}^{\cdot }$ radicals may recombine forming $\hbox {H}_2\hbox {O}_2$ (reaction 5b).

Upon introducing $\hbox {H}_2\hbox {O}_2$ to PB, the signal recorded during the pulsing period is slightly elevated, which reflects the increased concentration of $\hbox {HO}_2^{\cdot }$ by electrooxidation of $\hbox {H}_2\hbox {O}_2$ (reaction 10)^88^, additionally to water electrooxidation. Following the deprotonation of $\hbox {HO}_2^{\cdot }$ (reaction 11), such formed $\hbox {O}_2^{\cdot -}$ and electrogenerated $\hbox {HO}^{\cdot }$ catalyze the decomposition of $\hbox {H}_2\hbox {O}_2$ (Haber-Weiss reactions, 12 and 13)^83,89^, affecting the distribution of ROS and potentially also observed chemiluminescence. For PB and PB$/\hbox {H}_2\hbox {O}_2$, the luminescence signal is relatively constant in the pulsing period, implying a constant rate of water and $\hbox {H}_2\hbox {O}_2$ electrooxidation and follow-up reactions, with the overall process being controlled by the migration of ions constituting PB to respective electrodes. Elevated and retained (time independent) chemiluminescence observed in the presence of $\hbox {H}_2\hbox {O}_2$ implies that 1 s gaps inserted between consecutive pulses in the PEF seem to be sufficient for the regeneration of depleted $\hbox {H}_2\hbox {O}_2$ at the anode by its diffusion from the bulk of the liquid phase.

For BSA in the absence of $\hbox {H}_2 \hbox {O}_2$, the signal obtained in the pulsing period is higher than that recorded for PB, confirming that reactions involving BSA molecules lead to chemiluminescence. In the pH-neutral solution as used in this work, BSA is negatively charged^58^ and balanced by cations such as sodium or potassium. In the pulsing period, BSA is attracted to the anode, which causes its local preconcentration. Based on the BSA mobility at a given pH and ionic strength ($1.4\times 10^{-8} \, \hbox {m}^{2}\, \hbox {s}^{-1} \hbox {V}^{-1}$)^58^, one can calculate that upon thirty 100 $\upmu$s pulses, BSA molecules are moved by approximately 31 $\upmu$m (within each pulse, certain amount of BSA reaches the anode surface and does not move further). For the 0.6 mM solution, this length corresponds to the final amount of accumulated BSA of $1.8\times 10^{-5}$ mol  $\hbox {m}^{-2}$. To provide microscopical insight into this result, we further calculate the surface concentration in the monolayer of BSA molecules. This is $4.0\times 10^{-8}$ mol$\, \hbox {m}^{-2}$, assuming densely hexagonally packed spherical particles with a diameter of the BSA molecule (7.0 nm)^90^. The final amount of BSA accumulated at the anode surface thus corresponds to the molecular assembly with hundreds of layers, implying that BSA is available in large quantities and may be electrooxidized during PEF. In the pH neutral environment, this electrooxidation is, as other anodic processes (reactions 1 to 4 and 10), accompanied by a proton abstraction^91^, forming radicals in amino acid residues constituting the BSA molecule cumulatively denoted as $\hbox {BSA}^{\cdot }$ (reaction 14). Additionally, $\hbox {BSA}^{\cdot }$ may be generated in the diffusion layer of the anode upon the collision of BSA with $\hbox {HO}^{\cdot }$ (reaction 15) as the most reactive ROS^80^. The susceptibility of individual amino acids to the attack by $\hbox {HO}^{\cdot }$ is discussed further in this work.

The most probable further fate of $\hbox {BSA}^{\cdot }$ is its reaction with $^3\hbox {O}_2$^49,84,92^ which is naturally dissolved in water and also originates from reactions 4, 5a and 6 to 9, forming peroxylradical $\hbox {BSAOO}^{\cdot }$ (reaction 16). $\hbox {BSAOO}^{\cdot }$ undergoes subsequent reactions, either intramolecular cyclization or dimerization, both followed by a fragmentation (cumulatively denoted as reaction 17, see Vahalová et al.^49^ for detailed reaction mechanism) producing two kinds of emitters, electron-excited triplet carbonyls $^3$(R$'$COR$''$)*^93^ in either aldehyde or ketone moieties (emission by reaction 18) and $^1\hbox {O}_2$^94,95^. We cannot exclude that some other electron-excited molecular species are produced by the electrochemical excitation in our water-based system and contribute to the luminescence as the photon emitters. For example, it is known that other molecules present in our reaction scheme, such as $\hbox {HO}^{\cdot }$ have transitions between the electronic levels that can emit photon in visible range and can be produced in excited state in corona discharge (gas phase)^96^ but there is no evidence for them so far being produced in liquid phase in our experimental conditions (no discharge).

During the pulsing period, observed chemiluminescence increases with time (not observed for PB and PB$/\hbox {H}_2\hbox {O}_2$), see Fig. 3A, implying that the rate of emissive reactions increases. This is in accord with the notion of gradual accumulation of negatively charged BSA molecules at the anode surface upon each pulse. We note that the $\hbox {HO}^{\cdot }$ is an extremely reactive species, with involved reactions (5a, 5b, 6, 13 and 15) having diffusion-limited kinetics. The lifetime of the $\hbox {HO}^{\cdot }$ is a few microseconds corresponding to the diffusional length scale of tens to hundreds of nanometers^84^. The $\hbox {HO}^{\cdot }$ electrogenerated at the anode is thus swiftly converted to other less reactive species before it can diffuse to the bulk of the sample. Such circumstances corroborate the role of the migrative BSA accumulation at the anode in the observed chemiluminescence signal increase during the pulsing period. Low remanent chemiluminescence observed in the post-pulsing period is attributed either to (A) slow reaction of relatively stable $\hbox {O}_2^{\cdot -}$ with $\hbox {H}_2\hbox {O}_2$^83,89,97,98^ (both electrogenerated during the pulsing period by reactions 3, 5b, 7 and 11) to $\hbox {HO}^{\cdot }$ (reaction 12) which further reacts with BSA (reaction 15) or (B) slow (kinetically or sterically limited) steps within the BSA oxidation (reaction 17).

Upon introducing $\hbox {H}_2\hbox {O}_2$ to BSA, chemiluminescence was dramatically increased in all three experiment periods. In the pre-pulsing period, we attribute an elevated signal to the formation of $\hbox {HO}^{\cdot }$ and $\hbox {HO}_2^{\cdot }$ from $\hbox {H}_2\hbox {O}_2$ in a Fenton reaction (reactions 19 and 20) catalyzed by iron and copper ions in BSA^99^ (serum albumins serve in living organisms as carriers of metallic cations including iron^100^ and copper^101^). We note that the chemiluminescence increase noticed upon introducing $\hbox {H}_2\hbox {O}_2$ to BSA is significantly more pronounced compared to the increase observed after adding $\hbox {H}_2\hbox {O}_2$ to PB, pointing to much higher catalytic activity of metallic ions involved in the structure of BSA compared to those originating from the anode. In our previous contribution^49^, we analyzed solid BSA for total iron content, obtaining the value of 6 ± 3 ppm, which, for 40 mg/mL solution of BSA used in our current work, translates to 4 ± 2 $\upmu$M of iron. Such generated $\hbox {HO}^{\cdot }$ species boost reaction 15, which leads to more $^3$(R$'$COR$''$)* and $^1\hbox {O}_2$ emitters ultimately intensifying chemiluminescence. Additionally, $\hbox {HO}^{\cdot }$ and $\hbox {HO}_2^{\cdot }$ produced in the Fenton reaction may undergo reactions 5a, 6 and 7, which also generate $^1\hbox {O}_2$. We attribute slow chemiluminescence decay in the pre-pulsing period to the gradual consumption of $\hbox {H}_2\hbox {O}_2$ by the Fenton reaction. During the pulsing period, $\hbox {HO}^{\cdot }$ and $\hbox {HO}_2^{\cdot }$ are, additionally to the Fenton process, generated at the anode surface by electrooxidation of water and $\hbox {H}_2\hbox {O}_2$ (reactions 1, 3 and 10), while BSA molecules are accumulated at the anode. These processes synergistically contribute to the oxidation of BSA, as evidenced by a significantly enhanced chemiluminescence observed in the pulsing period for the BSA/$\hbox {H}_2\hbox {O}_2$ combination compared to the sum of chemiluminescence of individual BSA and $\hbox {H}_2\hbox {O}_2$ systems. The chemiluminescence signal increase is gradually slowed down, most likely with $\hbox {H}_2\hbox {O}_2$ concentration at the anode surface being the rate-limiting factor in the BSA oxidation. A significant residual chemiluminescence signal observed in the post-pulsing period above the baseline (pre-pulsing) level is, similarly as discussed above, attributed either to the BSA oxidation by $\hbox {HO}^{\cdot }$ formed from $\hbox {H}_2\hbox {O}_2$ and $\hbox {O}_2^{\cdot -}$ (reaction 12) electrogenerated previously during the pulsing period, or to slow (kinetically or sterically limited) steps within the BSA oxidation (reaction 17).

For all four inspected systems (PB, PB$/\hbox {H}_2\hbox {O}_2$, BSA and BSA$/\hbox {H}_2\hbox {O}_2$), we further integrated the luminescence signal in each of the three periods, taking a common time basis of 30 s (hatched areas in Fig. 3A). Obtained integrals are collectively plotted in Fig. 3B, clearly confirming the above-mentioned synergic effect. Important processes leading to intensified chemiluminescence in the BSA/$\hbox {H}_2\hbox {O}_2$ system are summarized in Fig. 3C.

It is important to mention that the charge transfer through the anode/electrolyte interface may be, besides the electrooxidation of water, $\hbox {H}_2\hbox {O}_2$ or BSA, also realized by the electrooxidation of the anode material (steel in this work). This process leads either to the passivation of the anode surface by (hydrated) oxide layers or to the dissolution of metallic cations to the liquid phase. We carefully inspected these phenomena. The surface of the employed anode was examined by optical microscopy before and after PEF treatment, with no visible changes observed. Additionally, we recorded the impedance spectrum of the chamber filled with the respective sample before and after the PEF treatment, observing no statistically significant changes in resistive and capacitive contributions. We further employed inductively coupled plasma optical emission spectroscopy (ICP-OES) to determine the amount of iron, chromium, manganese, and nickel (components of employed steel) in PB treated by PEF, with found signals being less than the detection limit of the employed device (0.02 mg$\, \hbox {L}^{-1}$). This limit represents roughly 2 % of the amount predicted by Faraday’s law considering the observed current and PEF profile, corroborating that PEF, as performed in this work, does not cause noticeable leaching of metallic cations into the liquid phase.

To resolve relative contributions of the suggested $^1\hbox {O}_2$ and $^3$(R$'$COR$''$)* emitters to the observed chemiluminescence signal, we further repeated the PEF treatment for PB$/\hbox {H}_2\hbox {O}_2$ and BSA$/\hbox {H}_2\hbox {O}_2$ with originally inspected 300–650 nm emission wavelength range being split to 300–550 nm and 550–650 nm intervals, by successively applying short-pass and long-pass filters. $^1\hbox {O}_2$ emits (via bimolecular decay) at 634 nm, whereas $^3$(R$'$COR$''$)* emits between 350 nm and 500 nm^59^. Such obtained luminescence transients were corrected for the averaged quantum efficiency of the employed photomultiplier in the respective wavelength range and are presented in Fig. SI 1-3. For PB$/\hbox {H}_2\hbox {O}_2$, as expected, virtually all observed luminescence was in the long wavelength range, corresponding to $^1\hbox {O}_2$ emission. For BSA$/\hbox {H}_2\hbox {O}_2$, a part of luminescence was in the short wavelength range, i.e. due to $^3$(R$'$COR$''$)*. Luminescence in the short wavelength range was observed in pre-pulsing as well as pulsing period, confirming that both oxidation of BSA initiated by $\hbox {HO}^{\cdot }$ from the Fenton reaction and ROS electrogenerated by PEF lead to $^3$(R$'$COR$''$)*. The luminescence was also increased in the long wavelength range, confirming that BSA also boosts the $^1\hbox {O}_2$ emission. We attribute this observation to the contribution of the tetraoxide pathway in the BSA oxidation (see^59^ for detailed reaction mechanism) as well as intensified bimolecular collisions among $\hbox {HO}^{\cdot }$ and $\hbox {HO}_2^{\cdot }$ as consequences of the Fenton reaction catalyzed by iron ions bound to the BSA molecule.

### Chemiluminescence at varied concentrations of the prooxidant

To explore the oxidation of BSA at varied amounts of electrogenerated ROS, we performed PEF treatment and chemiluminescence measurements at different concentrations of initially introduced $\hbox {H}_2\hbox {O}_2$ used as the prooxidant, (0.1 mM, 1 mM, and 10 mM), see Fig. 3D-G. Such $\hbox {H}_2\hbox {O}_2$ concentrations are well supra-physiological and the lower bound of our interval corresponds to values relevant for wounds^72,102^ and accelerated aging^103^. To obtain baseline behavior, we first performed the study in the PB system, with results shown in Fig. 3D. The averaged luminescence transient obtained for 0.1 mM $\hbox {H}_2\hbox {O}_2$ is indistinguishable from that recorded in the absence of $\hbox {H}_2\hbox {O}_2$ implying that the rate of its electrooxidation (reaction 10) at this concentration level is insufficient to generate a measurable chemiluminescence response. For 1 mM and 10 mM, luminescence is clearly increased in the pulsing period. For 10 mM, an elevated signal may also be noticed in the pre-pulsing and post-pulsing period, which we attribute to a non-electrochemical decomposition of $\hbox {H}_2\hbox {O}_2$ catalyzed by the anode surface containing (oxidized) iron atoms, which includes $\hbox {HO}^{\cdot }$ and $\hbox {HO}_2^{\cdot }$ as intermediates (see Lin et al.^104^ for detailed reaction mechanism). For all three inspected $\hbox {H}_2\hbox {O}_2$ concentrations, the luminescence signal upon pulsing quickly reverts to respective pre-pulsing values, which agrees with a short lifetime of $^1\hbox {O}_2$ due to monomolecular decay and bimolecular reactions (see Table SI 1-1 ﻿in SI 1 for list of rate constants of reactions considered in this work). For BSA$/\hbox {H}_2\hbox {O}_2$, a well-resolved chemiluminescence signal was observed (Fig. 3F), scaling with $\hbox {H}_2\hbox {O}_2$ concentration in all three periods of the experiment. This confirms the involvement of the Fenton process (reactions 19 and 20), electrooxidation of $\hbox {H}_2\hbox {O}_2$ (reaction 10) and subsequent conversion of $\hbox {HO}_2^{\cdot }$ to $\hbox {HO}^{\cdot }$ (reactions 11 and 12), which initiates the BSA oxidation (reactions 15 to 18).

Figure 3E,G shows integrals of luminescence signal for PB/$\hbox {H}_2\hbox {O}_2$ and BSA/$\hbox {H}_2\hbox {O}_2$ systems evaluated in all three periods (employing the common time basis of 30 s) as a function of $\hbox {H}_2\hbox {O}_2$ concentration. In all cases, observed dependences are non-linear, reflecting the complex mechanism of reactions preceding the emission. This also suggests that the rate of $\hbox {H}_2\hbox {O}_2$ electrooxidation is, under given conditions, limited by the number of catalytically active sites available on the anode surface (similarly as the rate of enzymatic reaction is limited by enzyme concentration in the Michaelis-Menten model) rather than by the concentration of $\hbox {H}_2\hbox {O}_2$ and its (diffusional) mass transport to the anode.

### Chemiluminescence in the presence of antioxidant enzymes

**Fig. 4:**
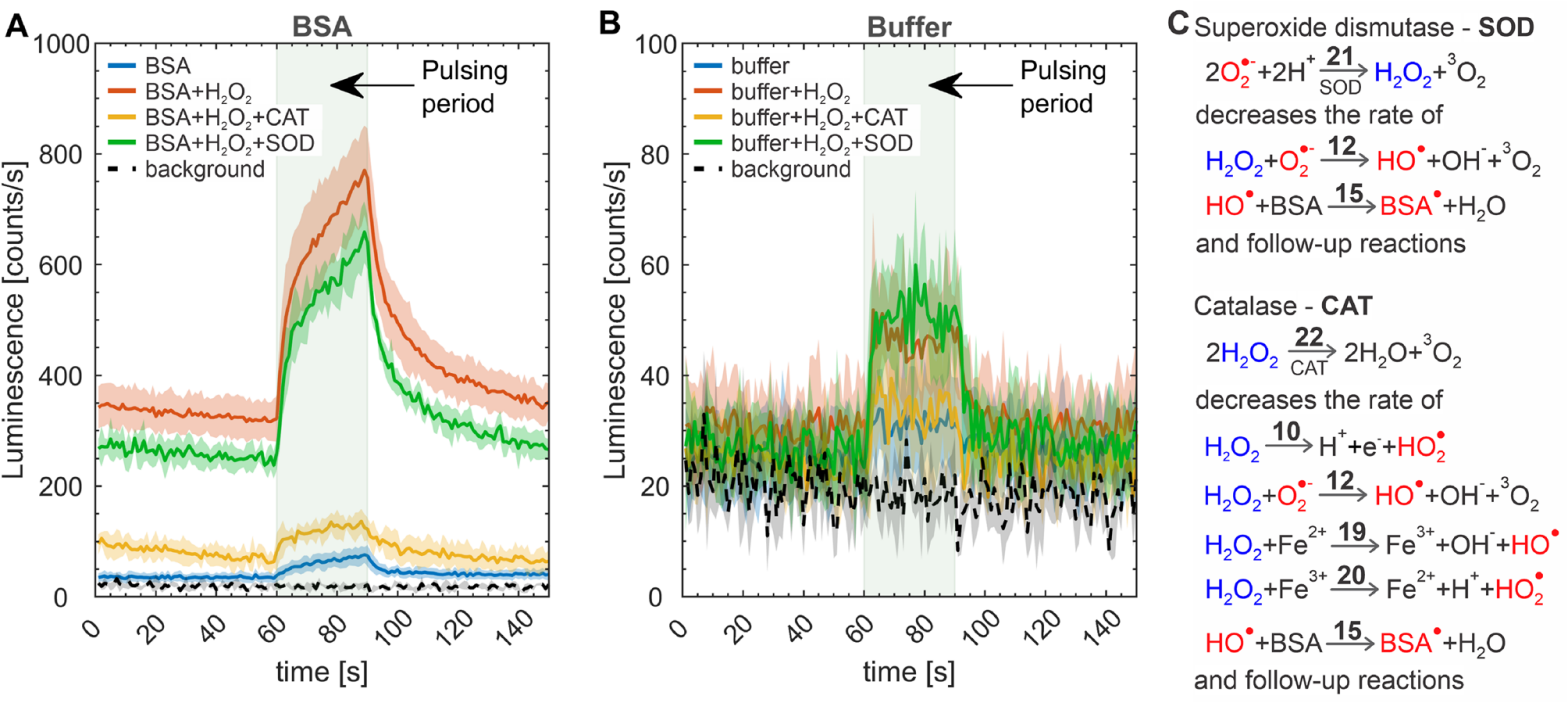
Averaged luminescence transients obtained in PEF experiments performed with (**A**) BSA and (**B**) PB in the absence and presence of $\hbox {H}_2\hbox {O}_2$ and with additionally introduced antioxidant enzymes (CAT or SOD). (**C**) A summary of important reactions modulating the chemiluminescence response of the BSA/$\hbox {H}_2\hbox {O}_2$ system by SOD and CAT.

We further explored the influence of antioxidant enzymes, namely SOD and CAT, on chemiluminescence in PB$/\hbox {H}_2\hbox {O}_2$ and BSA$/\hbox {H}_2\hbox {O}_2$ systems. SOD converts superoxide anion $\hbox {O}_2^{\cdot -}$ (a deprotonated form of $\hbox {HO}_2^{\cdot }$) to $\hbox {H}_2\hbox {O}_2$ and $^3\hbox {O}_2$ (reaction 21). CAT decomposes $\hbox {H}_2\hbox {O}_2$ to water and $^3\hbox {O}_2$ (reaction 22). Figure 4 shows obtained luminescence transients for PB$/\hbox {H}_2\hbox {O}_2$ and BSA$/\hbox {H}_2\hbox {O}_2$ with additionally introduced SOD or CAT. Interestingly, for PB$/\hbox {H}_2\hbox {O}_2$, the addition of SOD does not influence observed luminescence. Most likely, $\hbox {HO}_2^{\cdot }$ electrogenerated in reactions 3 and 10 is swiftly converted by fast reactions 6 and 7 to $^1\hbox {O}_2$ before its deprotonated form can react with SOD. The addition of CAT to PB$/\hbox {H}_2\hbox {O}_2$ leads, as expected, to the decrease of chemiluminescence due to partial consumption of $\hbox {H}_2\hbox {O}_2$ by the enzymatic reaction, decreasing its availability for the electrooxidation. For BSA$/\hbox {H}_2\hbox {O}_2$, both enzymes decreased chemiluminescence. The decrease by CAT reflects diminished availability of $\hbox {H}_2\hbox {O}_2$ for its electrooxidation, ultimately slowing down rates of emissive processes (reactions 9 and 18). For SOD, we have further evaluated the difference of signals recorded in its absence and presence (Fig. SI 1-1), obtaining a more pronounced signal decrease during the pulsing period and at the beginning of the post-pulsing period. This implies that SOD converts $\hbox {O}_2^{\cdot -}$ not only generated in the Fenton process (reactions 20 and 11) but also that formed by $\hbox {H}_2\hbox {O}_2$ electrooxidation (reactions 10 and 11). A more pronounced decrease in the post-pulsing period indirectly confirms the presence of remanent $\hbox {O}_2^{\cdot -}$ and validates the importance of its conversion to $\hbox {HO}^{\cdot }$ (reaction 12) in the proposed reaction mechanism. A summary of important reactions modulating the chemiluminiscence response of the BSA/$\hbox {H}_2\hbox {O}_2$ system by SOD and CAT is presented in Fig. 4C. We further performed control experiments for PB and BSA in the absence of $\hbox {H}_2\hbox {O}_2$, with introduced CAT or SOD, (Fig. SI 1-2). In PB, the introduction of enzymes led to no significant changes in the luminescence response confirming that they do not produce their own response. For BSA without $\hbox {H}_2\hbox {O}_2$, the introduction of CAT and SOD had no significant effect on the course of chemiluminescence signal. This suggests that electrogenerated $\hbox {H}_2\hbox {O}_2$ and $\hbox {O}_2^{\cdot -}$ are turned to other species by uncatalyzed reactions before they can be enzymatically converted.

### A note on cathodic reactions

It is important to realize that in the PEF experiment, the charge is transferred also at the cathode/electrolyte interface, which causes chemical changes in the diffusional layer of cathode (upper part of Fig. 2). These reactions are electroreduction of water, producing hydrogen (reactions 23 and 24), electroreduction of dissolved oxygen to $\hbox {O}_2^{\cdot -}$ (reaction 25) and in turn to $\hbox {H}_2\hbox {O}_2$ (reactions 26 and 27) and electroreduction of $\hbox {H}_2\hbox {O}_2$ to $\hbox {OH}^-$ with $\hbox {HO}^{\cdot }$ as the intermediate (reactions 28 and 29). While these species, apart from hydrogen and $\hbox {OH}^-$, appear as reactants in processes leading to chemiluminescence discussed above, the geometrical arrangement of the employed PEF chamber guarantees that cathodically generated species do not contribute to the observed luminescence signal (the cathode side adjacent to the sample is facing away from the photodetector).

To check for the existence of bubbles formed from gaseous products (hydrogen on cathode and oxygen on anode) arising from water electrolysis, we carefully inspected both electrodes after PEF treatment. No bubbles were observed, implying that the amount of electrogenerated products did not exceed their respective solubility limits in water.

### Chemical and spectroscopic analysis of samples subjected to pulsed electric field

**Fig. 5:**
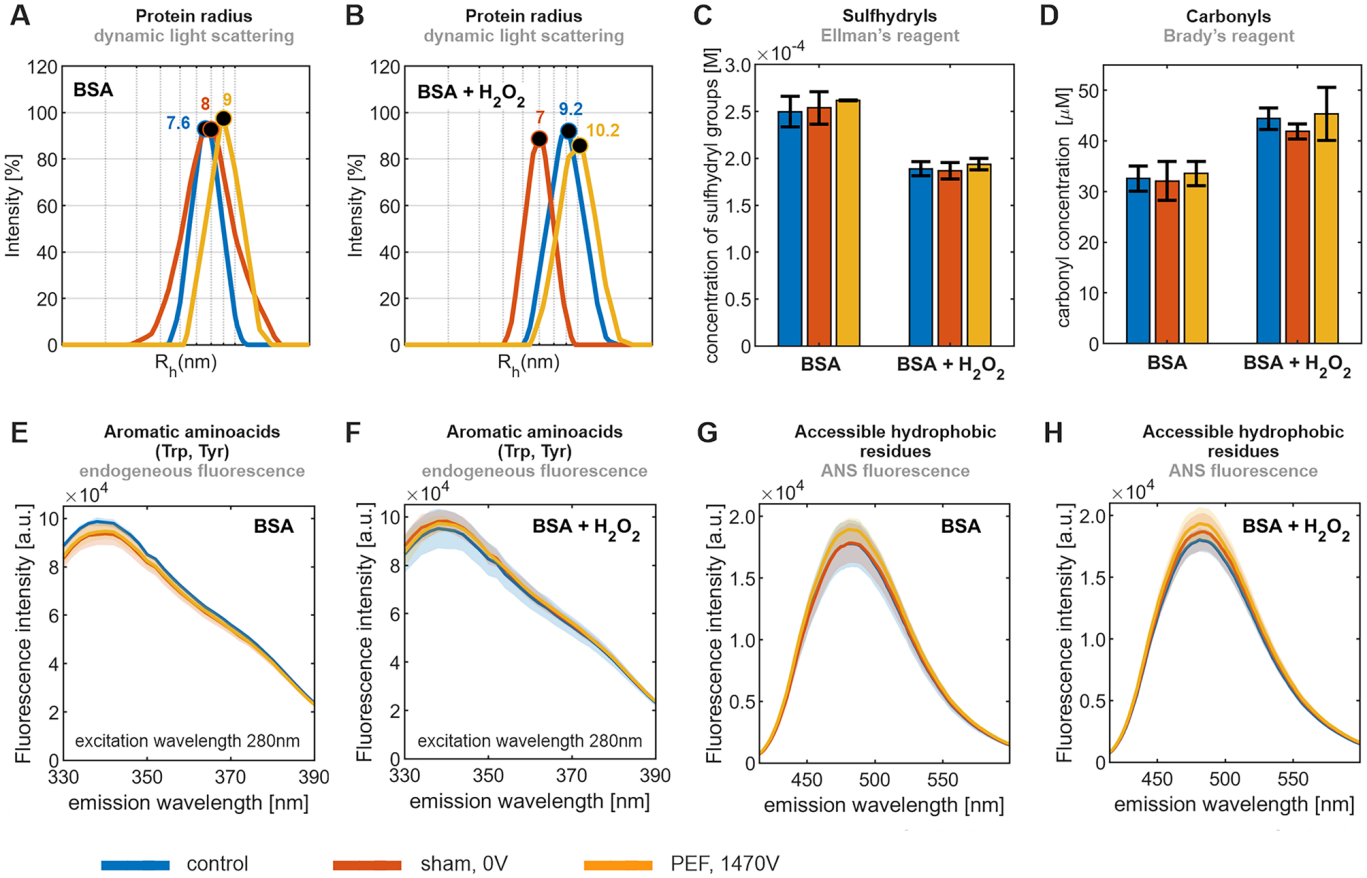
Results of biophysical and biochemical analyses performed for BSA and BSA/$\hbox {H}_2\hbox {O}_2$ samples subjected to PEF (orange), sham (dark red) and control (blue): averaged distribution of effective $\mathrm {R_h}$ values (**A**), (**B**), content of free sulfhydryl groups (**C**), content of carbonyl (aldehyde and ketone) groups (**D**), fluorescence spectra of samples excited at 280 nm (**E**), (**F**) and fluorescence spectra of ANS introduced to samples excited at 370 nm (**G**), (**H**).

To localize structural changes in the BSA molecule in samples treated by PEF either in the absence or presence of $\hbox {H}_2\hbox {O}_2$ as described above, we further performed a series of their biochemical and biophysical analyses, see Fig. 5. To evaluate the net effect of PEF, we additionally inspected respective samples not introduced to the PEF chamber (control) and samples introduced to the chamber, but with no PEF applied (sham).

Dynamic light scattering measurements were performed to evaluate relative changes of the size of the BSA molecule. Without $\hbox {H}_2\hbox {O}_2$ (Fig. 5A), PEF was found to have no profound effect. Introducing $\hbox {H}_2\hbox {O}_2$ (Fig. 5B) ﻿led to a slight, but not significant, increase in the average effective hydrodynamic radius (by ca. 15%) compared to case in the absence of $\hbox {H}_2\hbox {O}_2$. We attribute the small observed shift to a partial unfolding or contribution of a small number of aggregates upon mild oxidation of BSA molecules.

Ellman’s reagent was applied to determine the amount of free sulfhydryl (-SH) groups (Fig. 5C). For BSA without $\hbox {H}_2\hbox {O}_2$, no statistically relevant differences between PEF treated and control/sham samples were found. The introduction of $\hbox {H}_2\hbox {O}_2$ to BSA led to approximately 25 % decrease of -SH concentration for PEF treated as well as for control/sham samples, implying that $\hbox {H}_2\hbox {O}_2$ and further ROS generated from $\hbox {H}_2\hbox {O}_2$ cause extensive oxidation of -SH groups.

Brady’s reagent was employed to determine the amount of carbonyl groups as parts of newly formed ketone and aldehyde structures as another marker of oxidative damage (Fig. 5D). PEF was found not to affect the carbonyl content, while the introduction of $\hbox {H}_2\hbox {O}_2$ led to significantly elevated (approximately by 35 %) amounts for all three inspected samples.

We have further measured spectral characteristics of fluorescence excited at 295 nm to determine tryptophan residues (data not shown) and at 280 nm to determine the sum of tryptophan and tyrosine residues (Fig. 5E,F). Fluorescence intensity and emission wavelength maxima were virtually the same for all inspected samples. This implies that the introduction of $\hbox {H}_2\hbox {O}_2$ to the bulk of the sample causes no observable oxidation of aromatic amino acid residues in the BSA molecule. While $\hbox {HO}^{\cdot }$ electrogenerated in PEF at the anode is highly likely to react with aromatic amino acid residues in BSA molecules (see Table SI 1-1 in SI 1 for rate constants), these reactions are spatially confined to the diffusion layer of the anode, which has thickness of only tens to hundreds of nm. The fluorescence measurements, which reflect the averaged response of the entire sample volume, therefore cannot reveal changes in such narrow reaction zone.

ANS (8-anilino-1-naphthalenesulfonic acid) was further introduced to samples as a fluorescence probe to sense structural changes in surface hydrophobic parts of the BSA molecule. Performing PEF in the absence of $\hbox {H}_2\hbox {O}_2$ increased signal by 6 % (Fig. 5G) and in the presence of $\hbox {H}_2\hbox {O}_2$ ﻿by circa 10 % (Fig. 5H) ﻿(the addition of $\hbox {H}_2\hbox {O}_2$ to BSA per se had no effect, for exact values of fluorescence intensity see Table SI 1-3, in SI 1), implying that PEF and H_2_O_2_ synergically lead to slight exposure of hydrophobic moieties to the surrounding environment.

Overall, the introduction of $\hbox {H}_2\hbox {O}_2$ to BSA caused a concentration decrease of free -SH groups and elevated content of carbonyl moieties, both confirming oxidative damage. The effects of the PEF treatment on overall sample characteristics were minor. This observation is ascribed to the confinement of electrogenerated highly reactive ROS ($\hbox {HO}^{\cdot }$) at the anode surface limiting its impact on the sample bulk (chemiluminescence observed after PEF treatment was, as discussed above, ascribed to reactions involving significantly less reactive $\hbox {O}_2^{\cdot -}$ and various BSA radical species). Fluorescence analysis demonstrated that aromatic amino acid residues (tyrosine and tryptophan) and generally hydrophobic parts of the BSA molecule remained largely intact in all samples, suggesting that oxidative damage by $\hbox {H}_2\hbox {O}_2$ was located in hydrophilic (non-aromatic) regions of the BSA molecule. While this observation can seemingly be in a contradiction with the general notion that aromatic amino acid residues are more prone to the oxidation by ROS compared to their aliphatic counterparts^105^, one has to also consider varied accessibility of individual residues in the protein structure to short-living $\hbox {HO}^{\cdot }$. The combination of ANS fluorescence and dynamic light scattering measurements results suggested that oxidative damage by $\hbox {H}_2\hbox {O}_2$ led either to partial unfolding or slight aggregation of BSA molecules.

While the detailed analysis of the BSA oxidation at the level of individual amino acid residues involved in its structure is beyond the scope of this paper, we briefly point to differences in their reactivity with the $\hbox {HO}^{\cdot }$ radical. Rate constants between essential amino acids and the $\hbox {HO}^{\cdot }$ radical are listed in the SI 1 (Table SI 1-1). The primary factors determining reactivity are the chemical structure of the amino acid side chains and their accessibility within the protein’s three-dimensional structure^106,107^. Aromatic residues (*tyrosine*, *phenylalanine*, *tryptophan*, *histidine*) are highly susceptible to the radical attack due to their conjugated structure, which can undergo hydroxylation, ring-opening or form cross-links, such as dityrosine in tyrosine. *Arginine* is an aliphatic amino acid, which has reactivity comparable to aromatic amino acids, ascribed to its delocalized guanidino tail that forms a hydroxyl or carbonyl derivative upon oxidation. Sulfur-containing aliphatic residues (*cysteine* and *methionine*) are also prone to radical attack at rates comparable to aromatic residues. Cysteine’s thiol group can be oxidized to sulfinic/sulfonic acids or to form disulfides, crucial for protein folding and stability, while methionine oxidation leads to sulfoxide, sulfon or aldehyde, which can affect protein function. Purely aliphatic alkyl residues are less reactive than sulfur or aromatic residues, but can still be oxidized to hydroxyl or carbonyl derivatives. Branched chains (*leucine*, *isoleucine* and *valine*) are more reactive than linear ones (*alanine*, *glycine*). *Threonine* and *serine* are moderately reactive aliphatic amino acids, containing oxidizable hydroxyl groups, leading to side chain cleavage or carbonyl group formation. *Lysine* with comparable reactivity has a terminal amino group, which is converted to an aldehyde. *Proline*, as a special cyclic aliphatic amino acid, also has comparable reactivity and forms a wide variety of products including carbonyls. *Aspartate*, *glutamate* and their amide derivatives *asparagine* and *glutamine* belong to less reactive residues. Their radical attack is targeted to carboxylate/amide groups, leading to decarboxylation or other oxidative changes that affect protein charge and interactions.

In the case of BSA particularly, an earlier study employing Fenton reagents indicated^108^ that residues such as lysines, cysteines, arginines, prolines, histidines, and tyrosines are particularly prone to oxidative modifications, with early damage occurring near cysteine disulfide bridges. However, in that work^108^, while the molar ratio of oxidants to protein was similar, the treatment time was much longer than in our current experiments. That might cause differences leading, for example, to our situation where oxidation damage in tryptophans and tyrosines is not strong enough to be manifested in the changes of their fluorescence from bulk of the protein solution.

## Conclusion

This work has applied (bio)chemiluminescence sensing for the detection of electrogenerated reactive oxygen species, the oxidative processes of biomolecular solutions (taking bovine serum albumin as their representative), and how these processes are modulated by a prooxidant (hydrogen peroxide) and antioxidant enzymes (catalase and superoxide dismutase) as natural components of biological media. Our results suggest that the metal cations carried by the bovine serum albumin catalyze a Fenton-like decomposition of hydrogen peroxide to more reactive oxygen species, which in turn initiate oxidative damage of the protein. A significant increase of chemiluminescence was observed in the pulsed electric field, which we ascribed to the synergic effect of hydrogen peroxide electrooxidation and accumulation of bovine serum albumin at the anode surface due to electromigration. Biochemiluminescence measurements performed in judiciously selected spectral ranges indicated the involvement of singlet oxygen and electron-excited triplet carbonyl moieties as emitters, corroborating the correctness of the proposed reaction mechanism. We further show that catalase and superoxide dismutase antioxidant enzymes have protective effects, with the latter enzyme removing superoxide generated both in the Fenton reaction and hydrogen peroxide electrooxidation.

Our work demonstrates that (bio)chemiluminescence sensing is a powerful tool for detecting in-situ (electro)generated reactive oxygen species and for exploring their interactions with biomolecules. Comparison of signals recorded under open circuit conditions and applied voltage enabled revealing details in reaction mechanisms of interfacial charge transfer processes and follow-up reactions relevant to pulsed electric field applications. The sensing technique developed in this work is label-free, compatible with in operando conditions, and non-destructive to the bulk of the inspected sample. It enables a systematic control of the amount of electrogenerated reactive oxygen species by varying concentration of hydrogen peroxide used as a prooxidant. Our results provide new insights into the molecular action of pulsed electric field and thus have impact on bioelectrochemistry, pulsed electric field, bioelectromagnetics as well as bioelectronics and biophotonics research communities. While our work succeeded at getting insights into the interactions of proteins with reactive oxygen species electrogenerated in pulsed electric field in model systems, it did not take into account the matrix effects of complex real-world biological samples. Besides other types of biomolecules, these often include inorganic ions (sodium, potassium, chloride, hydrogen carbonate) at concentrations on the orders of 10 mM and 100 mM. These ions substantially increase the electric conductivity of samples and, hence, the formation rate of reactive oxygen species. Furthermore, the presence of chloride anion leads to the parallel generation of reactive chlorine species (mainly hypochlorous acid and its anion hypochlorite) at the anode, which also react with biomolecules. Exploring the effects of these system constituents will be critical to evaluate the overall influence of pulsed electric field on real-world samples.

## Methods

### Chemicals

Milli-Q water (Millipore, resistivity 18.2 M$\Omega \,$cm) was used to prepare all solutions and perform all cleaning procedures. Bovine serum albumin (BSA, heat shock fraction, lyophilized powder, Sigma - Aldrich, A3803-50G) was dissolved at a concentration of 40 mg$\, \hbox {mL}^{-1}$ (0.6 mM). The pH value of this solution was 7.2 ± 0.2, its conductivity was 0.033 ± 0.003 S $\hbox {m}^{-1}$. We chose phosphate buffer (PB) as a control, purely inorganic sample, with identical pH and conductivity as the BSA solution, comprising 0.612 mM $\hbox {NaH}_2\hbox {PO}_4$ and 1.411 mM $\hbox {Na}_2\hbox {HPO}_4$. Solid chemicals used in the preparation of PB were $\hbox {NaH}_2\hbox {PO}_4$ ·  2 $\hbox {H}_2$O (P-Lab, D 03102) and $\hbox {Na}_2\hbox {HPO}_4$  · 12 $\hbox {H}_2$O (P-Lab, H 08102). For selected experiments, hydrogen peroxide ($\hbox {H}_2\hbox {O}_2$, a non-stabilized 30 % aqueous solution, p.a., Penta, 23980-11000), was introduced to the sample (either PB or BSA) in final concentrations of 0.1 mM, 1 mM, and 10 mM. For selected experiments, catalase (CAT) from bovine liver (lyophilized powder, 2000–5000 units$\, \hbox {mg}^{-1}$ protein, Sigma - Aldrich, C9322-1G) was introduced to the sample in a final concentration of $3.2\times 10^{-5}$ mM. For selected experiments, superoxide dismutase (SOD) from bovine erythrocytes (lyophilized powder, $\ge$3000 units$\, \hbox {mg}^{-1}$ protein, Sigma - Aldrich, S7571-30KU) was introduced to the sample in a final concentration of $9.9\times 10^{-5}$ mM. These concentrations of CAT and SOD were selected empirically to ensure a measurable decrease in chemiluminescence of the BSA/$\hbox {H}_2\hbox {O}_2$ sample within the experimental time window. Our objective was to showcase the impact of CAT and SOD on chemiluminescence rather than quantifying the observed changes or relating them to the catalytic activity of CAT and SOD.

All prepared solutions were stored under ambient pressure and temperature in contact with lab air and hence additionally contained approximately 0.3 mM of dissolved oxygen.

The conductivity of solutions was measured employing SevenCompact S230 probe (InLab 751-4mm, Mettler Toledo) calibrated with a standard solution of 84 $\upmu$S$\, \hbox {cm}^{-1}$ (Hanna Instruments, HI7030) at a temperature of 25 $^\circ$C. The pH was determined using a pH meter (Orion Star A111, Thermo Scientific) calibrated with buffer solutions of pH 4.0, 7.0, and 10.0 (Carl Roth, A517.3, P713.3, and 8086.1, respectively).

### Pulsed electric field equipment and treatment

A specialized chamber for conducting the pulsed electric field (PEF) treatment on samples employed in this work (see Fig. 7A for cross-sectional image) was designed and crafted at the Institute of Photonics and Electronics at the Czech Academy of Sciences in Prague, Czechia. This chamber was machined from a block of the dielectric polymethylmethacrylate (PLEXIGLAS GS, Zenit); it features an anode at the bottom and a cathode at the top arranged in a thin-layer parallel-plate configuration (gap between electrodes of 2.00 mm). The anode is solid and manufactured from a plate of stainless steel (type 1.4571, thickness 6 mm, AKROS). The cathode is manufactured from a plate of stainless steel (type 1.4304, thickness 130 $\upmu$m, PragoBoard) and, to enable sensing of chemiluminescence, it contains hexagonally arranged drilled holes, with a diameter of 1.0 mm and a center-to-center spacing of 1.5 mm i.e. resulting in 50 % void area. Both cathode and anode have 26 mm diameter, defining the cross-sectional area of the liquid sample within the PEF chamber (area 5.31 $\hbox {cm}^{2}$). The PEF chamber volume is 1.06 mL.

**Fig. 6:**
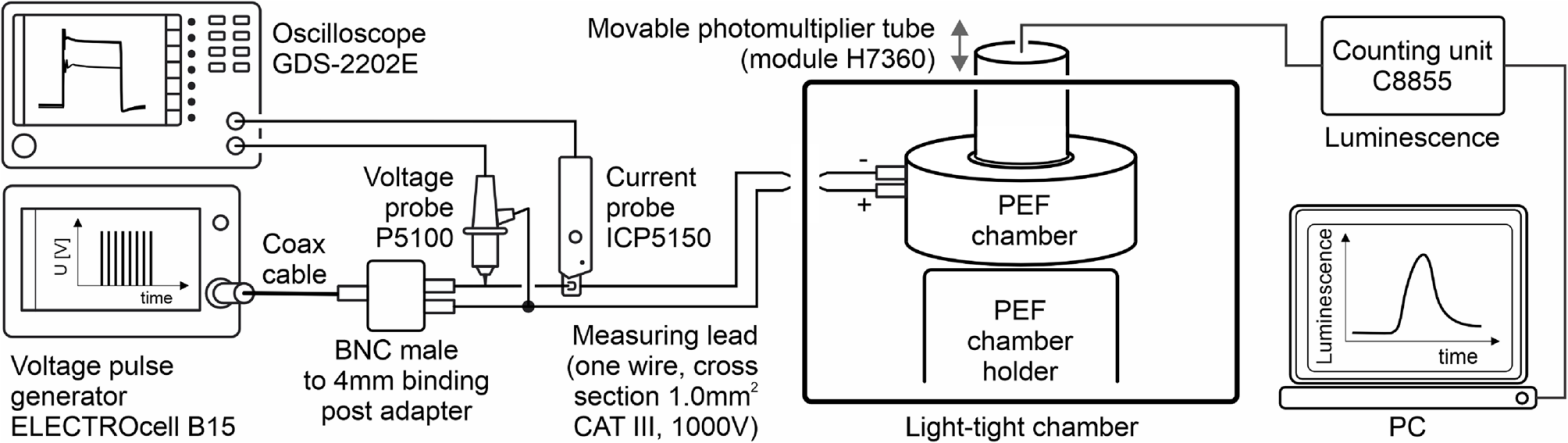
Scheme of the experimental platform (circuit) employed in this work to generate PEF, measure applied voltage and resulting current and luminescence signal.

**Fig. 7:**
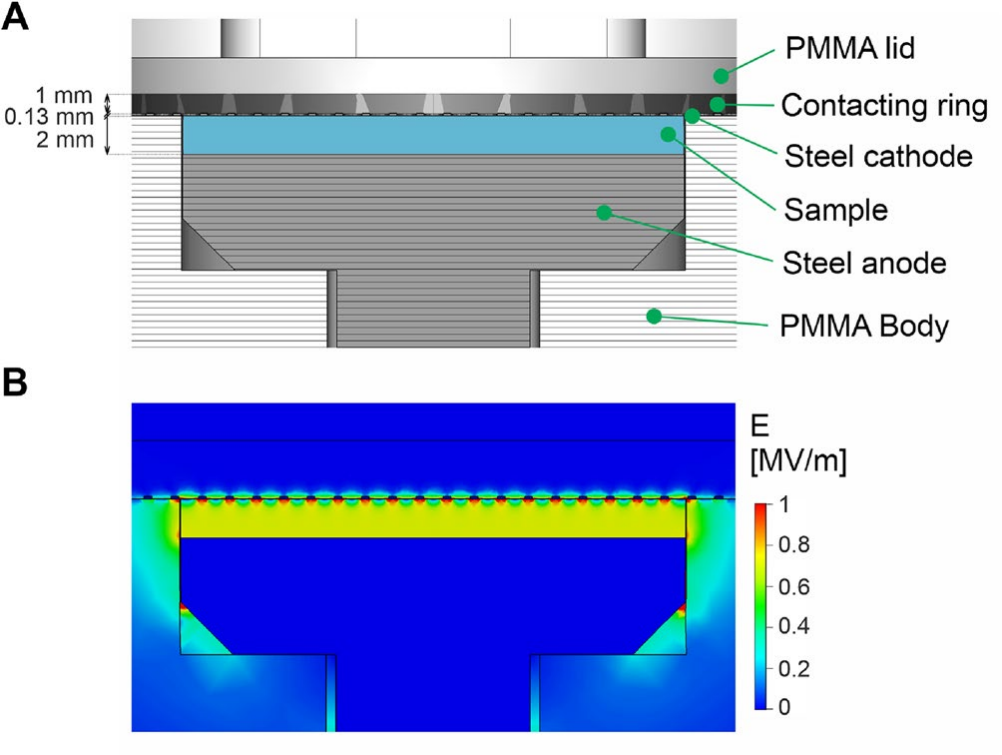
(**A**) Cross-sectional image of the PEF chamber employed in this work. (**B**) Distribution of electric field strength in the PEF chamber for applied voltage of 1470 V.

**Fig. 8:**
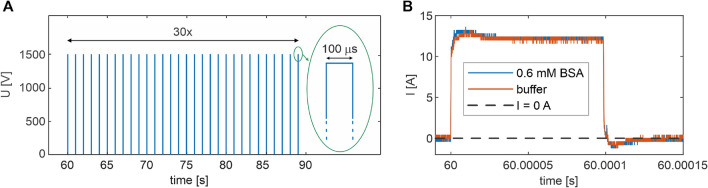
(**A**) PEF profile applied in this work. (**B**) Representative current transients obtained in the first pulse of PEF for PB and BSA sample.

**Fig. 9:**
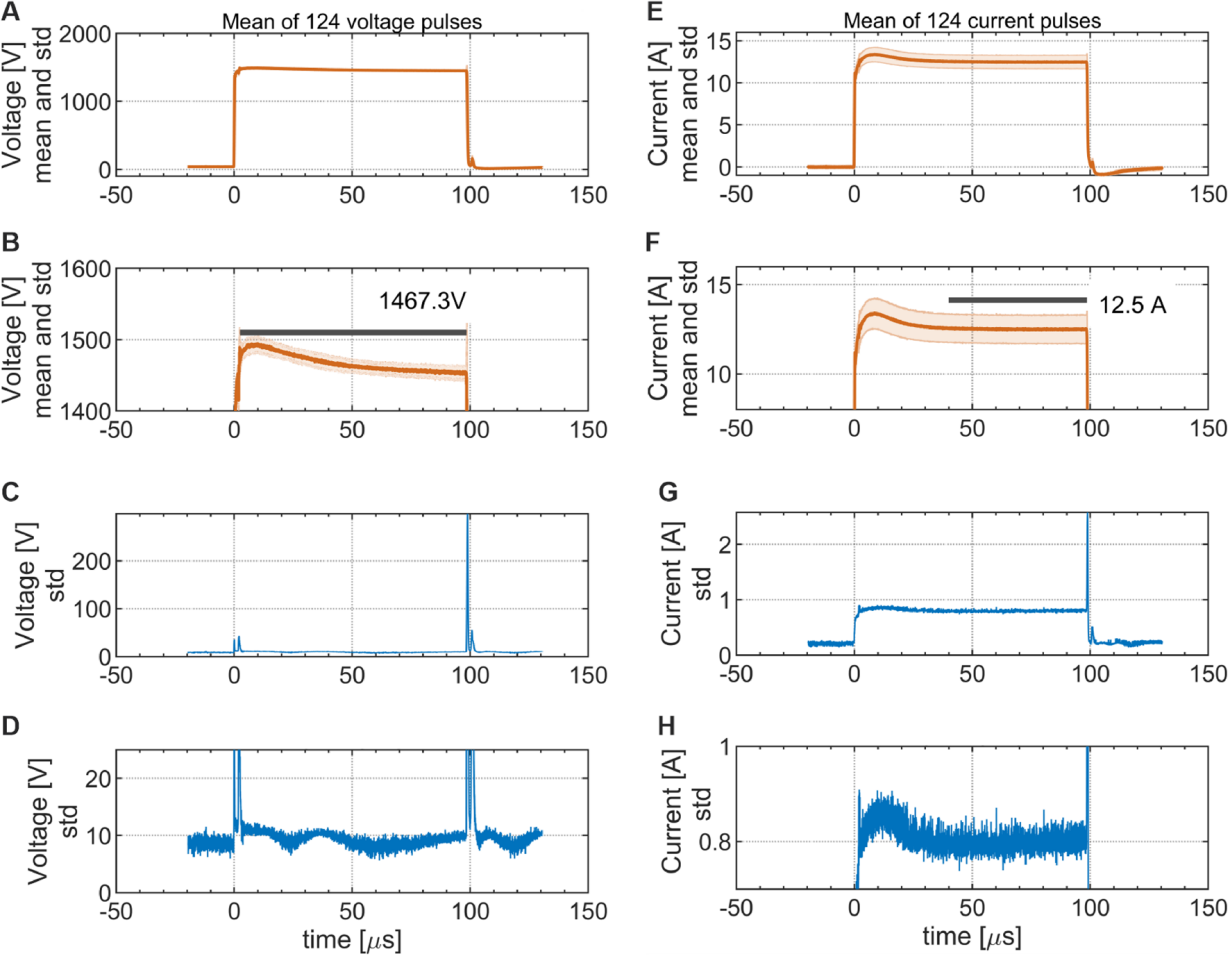
(**A**-**D**) Averaged measured characteristics of voltage profile applied in each pulse of PEF - mean value (**A**, close-up **B**) and standard deviation (**C**, close-up **D**). (**E**-**H**) Averaged measured resulting current transients - mean value (**E**, close-up **F**) and standard deviation (**G**, close-up **H**). This dataset was compiled from representative curves obtained for all samples inspected in this study.

The PEF was formed by the pulse generator (ELECTROcell B15, Leroy-Biotech), which also continuously recorded applied voltage and resulting current flowing between the anode and cathode. The characteristics of the initial pulse in each PEF sequence were independently recorded using high voltage (P5100, Durlclth) and current (ICP5150, Instrance) probes connected to the oscilloscope (GDS-2202E, GW Instek), see Fig. 6 for the measurement platform (circuit). PEF consisted of 30 unipolar pulses, each with voltage amplitude of 1470 V and a width of 100 $\upmu$s, fired at a frequency of 1 Hz (see Figs. 1B and 8A). For the employed PEF chamber, the applied voltage of 1470 V corresponds to the electric field strength of 0.735 MV$\, \hbox {m}^{-1}$. Figure 7B shows the distribution of electric field strength in the PEF chamber calculated in the CST Microwave Studio for applied 1470 V voltage. Figure 8B shows representative current transients obtained in the first pulse for PB and BSA samples. Figure 9 A-D depicts averaged measured characteristics of the voltage transient applied in each pulse, while Fig. 9E–H shows averaged measured resulting current transients﻿.

To obtain electric characteristics of inspected samples in the PEF chamber without a DC load, we further recorded their impedance spectra (IS, magnitude and phase shift) in the frequency range from 20 Hz to 300 000 Hz and using probing voltage with 0.5 V_rms_ amplitude employing an LCR meter (BK891, BK PRECISION). These measurements served for a triple purpose: (1) assessing the conductivity of the sample, (2) confirming the accurate assembly of the PEF chamber and (3) evaluating the action of PEF on the sample by performing IS measurements before and after the PEF treatment (IS data not shown in this work).

Upon each PEF experiment, the chamber and electrodes were briefly rinsed with water. Upon three consecutive PEF experiments, the cathode and the anode underwent a thorough cleaning process as follows. Both electrodes were immersed in 30 % $\hbox {H}_2\hbox {O}_2$ for 5 min; they were subsequently rinsed with water and dried in a stream of gaseous nitrogen (purity$\ge$99,999 %, Linde). Cleaning of the anode was followed by its mechanical polishing using an aqueous suspension of 0.3 $\upmu$m alumina supported by a microfiber cloth. To ensure the removal of any remaining alumina microparticles, the anode was rinsed with water and ultrasonicated in water for 5 min. This cleaning procedure was essential to obtain reproducible chemiluminescence data.

### Luminescence measurement equipment

The PEF chamber filled with the respective sample was situated within a light-tight black box designed and constructed in the Institute of Photonics and Electronics at the Czech Academy of Sciences in Prague, Czechia. This box incorporated a photomultiplier tube (PMT) module H7360-01, Hamamatsu Photonics K.K. mounted to the ceiling of the box. This PMT sensed photons within a spectral range from 300 nm to 650 nm, i.e. covering the emission wavelength of species relevant for this work. The PMT was connected to a counting unit (C8855, Hamamatsu Photonics K.K.), offering an interface for computer connection. The PEF chamber was positioned close to the PMT, maintaining a fixed distance of 8 mm between the PMT module and the cathode. Before actual experiments, background measurements of photon counts were performed, encompassing both empty box without the PEF chamber (“dark counts”) and the dry, empty PEF chamber installed in the box, with values stabilizing at 21 ± 6 counts$\, \hbox {s}^{-1}$. To distinguish the relative contributions of chemiluminescence emitters, we employed a shortpass filter (84-708, Edmund Optics) and a longpass filter (62-984, Edmund Optics), with a cut-off wavelength of 550 nm and a diameter of 25 mm. The filter holder dimensions were mechanically designed to pass photons from the sample to the detector only through the filter, if present, to avoid any unfiltered light leakage from the sides. To quantitatively assess chemiluminescence contributions (Fig. SI 1-3), we considered the quantum efficiency (QE) of the chosen PMT in respective wavelength-resolved measurements. QE, representing the percentage of incident photons effectively converted into photoelectrons emitted from the photocathode of the PMT, varies with the wavelength of the incident light. This characteristic was necessary for converting measured photon counts to correct chemiluminescence magnitude. The average QE for the wavelength range of 300 nm to 550 nm was 12.2 %, while for the range of 550 nm to 650 nm, it was 0.55 %, with these values being used in the conversion.

### Timing of the experimental procedures

Maintaining precise timing in the preparation of samples (namely adding $\hbox {H}_2\hbox {O}_2$, CAT, and SOD to BSA and PB) and in transporting such prepared samples to the PEF chamber was found to be crucial to achieve the reproducibility of realized experiments, as it influenced the immediate chemiluminescence response of samples due to fast kinetics of involved chemical reactions. Due to the time required for the PEF chamber assembly and carrying out IS measurements, it was not technically possible to monitor chemiluminescence signals immediately after the sample preparation.

**Fig. 10:**
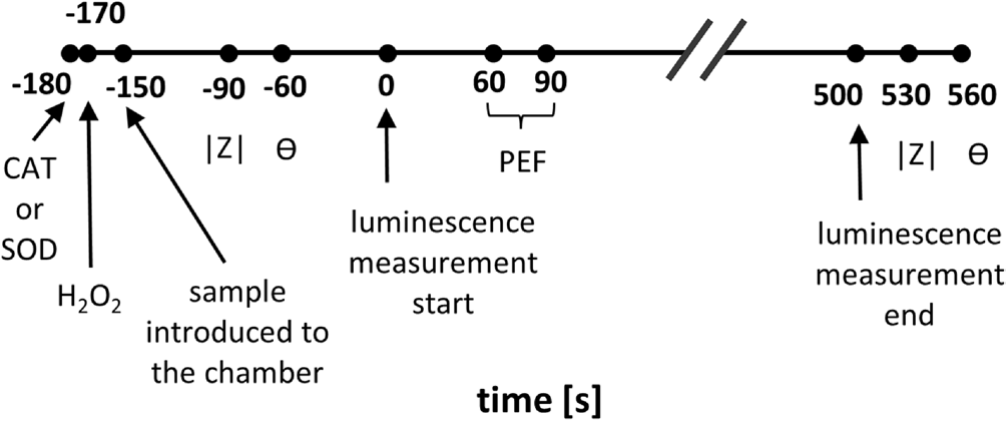
Timing scheme followed in the preparation of samples and subsequent PEF experiments. Z and $\Theta$ stand for the impedance magnitude and phase shift measured by LCR meter, respectively.

In the following description, time t = 0 s refers to the point at which luminescence monitoring was commenced (see Fig. 10). For samples containing either CAT or SOD, the respective enzyme was added to the sample in an Eppendorf tube at − 180 s, and the resulting mixture was briefly vortexed. For samples containing $\hbox {H}_2\hbox {O}_2$, this constituent was added to the sample in an Eppendorf tube at − 170 s, and the resulting mixture was briefly vortexed. At − 150 s, a respective sample was introduced to the open PEF chamber (cathode demounted). In particular, the volume of 1.24 mL was introduced, which is slightly more than the volume of the PEF chamber (1.06 mL). Upon mounting the cathode to the PEF chamber, an excess portion of the sample was forced to move through the cathode perforation, ensuring the absence of air bubbles in the interior of the PEF chamber. The chamber was subsequently placed in the light-tight box, connected to the LCR meter, and the impedance spectra (magnitude Z and phase shift $\Theta$) were measured around − 90 s and − 60 s. Luminescence was monitored from 0 to 500 s. In the period between 0 and 60 s, no PEF was applied to electrodes, allowing background signal to be collected (pre-pulsing period). Between 60 s and 90 s, PEF was applied (pulsing period) with the profile as described above (Figs. 1B and 8A), with an electric current being recorded. For 90 s to 500 s, no PEF was applied (post-pulsing period), while luminescence was still recorded to evaluate the remanent signal. Significant changes in the chemiluminescence were observed only up to 150 s and, therefore, only this time period is presented in figures and discussed in the manuscript. The impedance spectra (Z and Θ﻿) were measured again around 530 s and 560 s.

### Monitoring temperature in the PEF experiment

**Figure 11:**
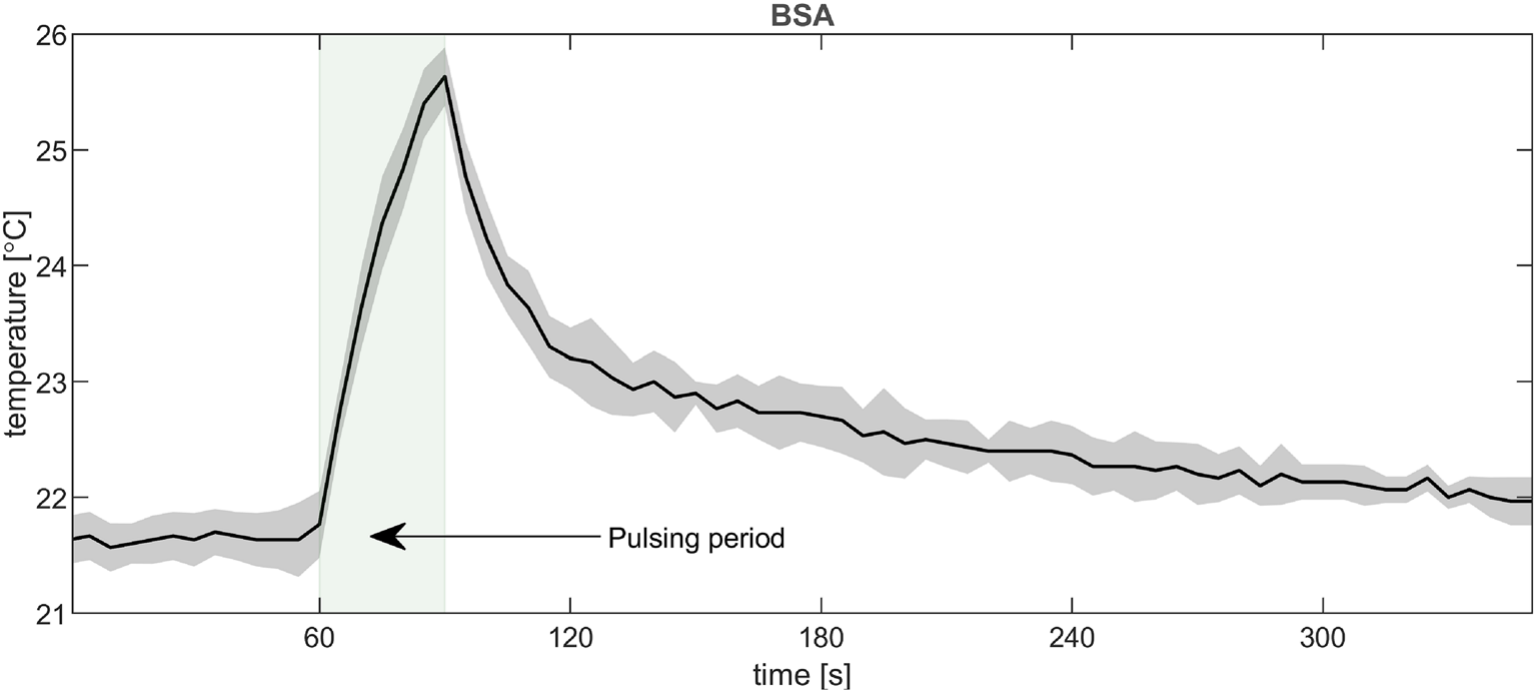
Evolution of temperature in the PEF experiment with BSA sample as measured by a thermal IR camera directed to a black opaque adhesive tape being in direct contact with the cathode.

PEF experiments were performed at ambient pressure and initial temperature of 22 $^\circ$C. The temperature of the PEF chamber was monitored in selected experiments using a thermal IR camera (Therm-App, Opgal Optronic Industries) in conjunction with a smartphone running the Therm-App Plus application. In particular, the camera sensor was directed to a black opaque adhesive tape in direct contact with the cathode. Prior to experiments, calibration of the camera sensor was conducted using a reference thermometer (SuperFast ThermoJack PRO, Dostmann, 5020-0552) as follows. The tape was affixed to a glass beaker filled with water of varied temperature (either 23 $^\circ$C or 42 $^\circ$C). The emissivity of the tape in the camera software was empirically adjusted so that the temperatures displayed by the IR camera corresponded accurately to the actual temperatures. Such found emissivity value was 0.6. A representative temperature transient obtained for the PEF experiment is shown in Fig. 11.

During the pulsing period, the temperature transiently increased, with the maximum of 26 $^\circ$C (i.e. by 4 $^\circ$C) at the end of the period. This increase is attributed to the Joule heating due to ionic migration in the sample induced by the PEF. Such subtle temperature increase is believed to have no substantial effect on the conformation of BSA and catalytic activity of SOD and CAT. The experimentally observed temperature increase of 4 $^\circ$C is much less compared to the theoretically predicted value (circa 12 ^∘^C) for Joule heating for the applied PEF profile and resulting current, which we attribute to an efficient heat flow from the solution via electrodes.

We have further monitored electric current within the pulsing sequence, typically obtaining an overall increase of 7 to 8%. Employing physical relationships between the observed current, sample conductivity, ionic mobilities and solution viscosity and the dependence of the latter on temperature, we could indirectly determine temperature changes in the PEF chamber during pulsing, obtaining an increase of ca 3 $^\circ$C (final temperature of 25 $^\circ$C), see SI 2. Employing Ohm’s law, we further estimated current in regions of the chamber with elevated electric field strength (just below the perforated cathode, see Fig. 7B). The upper estimate of the temperature increase (calculation not considering diffusive heat dissipation) is 12 $^\circ$C (final temperature of 34 $^\circ$C). This temperature is still within the stability range of proteins, excluding local thermally induced changes in the protein structure in this work. It is very likely that the temperature increase in the hotspots in the experiment is much lower than the maximum predicted here.

### Biochemical and biophysical analysis of BSA and BSA/$\hbox {H}_2\hbox {O}_2$ samples subjected to the PEF treatment

We conducted an additional series of analyses of BSA and BSA/$\hbox {H}_2\hbox {O}_2$ samples subjected to PEF treatment as follows. Our investigation also included BSA and BSA/$\hbox {H}_2\hbox {O}_2$ samples not introduced to the PEF chamber (control) as well as samples introduced to the PEF chamber but without applying PEF (sham, 0 V). Control samples were kept in Eppendorf vials for the entire experiment duration. Sham samples were kept in the PEF chamber for the entire duration of the experiment. The comparison of results obtained for control and sham samples allowed us to evaluate the influence of PEF chamber and electrode materials on BSA and BSA/$\hbox {H}_2\hbox {O}_2$ samples, with chemiluminescence also being recorded (data not shown).

#### Quantification of free sulfhydryl groups

The concentration of free sulfhydryl (-SH) groups was determined using Ellman’s reagent (5,5$'$-dithio-bis-(2-nitrobenzoic acid), DTNB, Thermo Scientific Chemicals, 22582). Following the protocol provided with the purchased reagent, we measured the absorbance at 412 nm to determine 2-nitro-5-thiobenzoic acid (TNB) as a product of the reaction between free –SH groups and the Ellman’s reagent. Ellman’s reagent was stored as 4 mg/mL solution in the reaction buffer (0.1 M $\hbox {Na}_3\hbox {PO}_4$, 1 mM EDTA, pH = 8.0, adjusted by HCl). These measurements were realized in a cuvette module (Spark, Tecan) employing single-use cuvettes (Brand, BR759200) with a 1 cm path length. Such obtained absorbance values were corrected for blank composed of reaction buffer with Ellman’s reagent. The final concentration of the Ellman’s reagent was 180 $\upmu$M and that of BSA was 54 $\upmu$M (3.6 mg/mL), all dilutions were made using the reaction buffer. Free ﻿–SH groups were quantified employing Lambert-Beer’s law and the molar extinction coefficient of TNB (value 14150 $\hbox {M}^{-1} \, \hbox {cm}^{-1}$).

#### Quantification of carbonyl groups

To determine the concentration of carbonyl groups (in aldehyde and ketone structures), Brady’s reagent (2,4-dinitrophenylhydrazine, DNPH, Protein Carbonyl Content Assay Kit, Sigma-Aldrich, MAK094-1KT) was employed, following the standard protocol outlined in the kit. The concentration of the hydrazone adduct formed between the carbonyl group and DNPH was determined by measuring absorbance at 375 nm employing a multiwell plate reader (Spark, Tecan) and 96 Well Clear Plate (path length of 0.2893 cm, MAK094E-1EA-KC), provided in the Assay Kit, and Lambert-Beer’s law considering molar extinction coefficient of the formed hydrazone adduct ($\varepsilon ^{mM}$ = 22 $\hbox {mM}^{-1} \, \hbox {cm}^{-1}$).

#### Tryptophan and tyrosine fluorescence

Aromatic amino acid residues, specifically tryptophan and tyrosine, underwent quantification by fluorescence spectroscopy measurements. For sample preparation, the respective samples were diluted by factor of 120, and 200 $\upmu$L of such diluted sample was placed into individual wells of a 96-well plate (Thermo Scientific, 265301). The subsequent fluorescence analysis was carried out using the multiwell plate reader (Spark, Tecan), with excitation wavelengths set to 280 nm (to determine the sum of tryptophan and tyrosine residues), and to 295 nm (to determine tryptophan residues) and a detected spectrum ranging from 330 nm to 400 nm.

#### Fluorescence with 8-anilino-1-naphthalenesulfonic acid probe

The presence of surface hydrophobic structures in the BSA molecule was assessed by introducing the fluorescent molecular probe 8-anilino-1-naphthalenesulfonic acid (ANS, Thermo Scientific Chemicals, J65538-06). ANS binds non-covalently to these moieties leading to the enhancement of its fluorescence intensity. ANS was stored as a 10 mM stock solution in DMSO as solvent. In particular, 100 $\upmu$M ANS (final concentration) was added to inspected sample diluted by pure water by factor of 240, i.e the final concentration of BSA would corresponded to 2.5 $\upmu$M (0.16 mg/mL). This dilution step was performed to assure that enough ANS is available for the sensing of hydrophobic structures within BSA and that the resulting fluorescence signal is within the dynamic range of the employed spectrometer. These concentration ranges were close to the ones in the previous studies^109,110^. Resulting mixtures were incubated for 40 minutes and then analyzed employing a 96-well plate (Thermo Scientific, 265301, 200 $\upmu$L of the mixture was introduced to each well). Fluorescence measurements were carried out using a multiwell plate reader (Spark, Tecan) at excitation wavelength 370 nm and the emission spectra were measured from 415 nm to 600 nm.

#### Dynamic light scattering

The effective hydrodynamic radius $\mathrm {R_h}$ of BSA molecules depending on the treatment as described above was measured by a dynamic light scattering (Brookhaven Instruments, 90 Plus PALS configuration, USA). 60 $\upmu$L of original sample was analyzed in disposable UV/VIS cuvettes (UVette 220–1600 nm, Eppendorf, 30106300) with 90$^\circ$ measurement angle. Experimental parameters were as follows: measurement mode: proteins; wavelength: 659 nm; cell (cuvette) type: BI-SM50; temperature: 22 $^\circ$C; set duration: 180 s; equilibration time: 120 s; expected particle size: 5–25 nm with dust filter; total measurements: 3; time interval: 5 s; liquid: water; refractive index of the sample (particles = proteins): 1.445; uniform spheres; protein concentration: 0.6 mM (40 mg/mL); baseline normalization: slope analysis; size distribution: Non-negative least squares algorithm; Threshold: 15.

#### Metal cations determination

The analysis of the metal content in the samples was performed by inductively coupled plasma optical emission spectrometry on device: ICP-OES Optima 8000 (Perkin Elmer) with a Scott spray chamber. Due to the low volume the samples were diluted 2$\times$. The emission intensities at several wavelengths for each element were analysed. The wavelength with high intensity and the least interference of the response with other elements was used for the evaluation, in particular: Ni (221.648 nm), Fe (239.562 nm), Cr (267.716 nm), and Mn (257.610 nm). The calibration curve method was used to determine the concentration. The calibration curve was constructed by analyzing known standards diluted from commercially supplied certified reference solutions from Analytika, Ltd. (Czechia). Due to the complex matrix and dilution of samples, the detection limit of analysis was estimated 0.02 mg$\, \hbox {dm}^{-3}$ for all elements analyzed. Under the mentioned level, it was not possible to distinguish between noise and analyte signal.

## Supplementary Information

Supplementary Information 1.

Supplementary Information 2.

## Data Availability

Raw data are available in the Zenodo database under DOI: 10.5281/zenodo.12542774.
