# Supplementary material for: Modulation of pulsed electric field induced oxidative processes in protein solutions by pro- and antioxidants sensed by biochemiluminescence
Source: Sci Rep. 2024 Sep 30;14:22649. doi: 10.1038/s41598-024-71626-6 (PMC11442601; doi:10.1038/s41598-024-71626-6)
Supplement: Supplementary file 1 — Supplementary Information 1. [file 41598_2024_71626_MOESM1_ESM.pdf]

Supplementary information 1 for ”Modulation of Pulsed Electric Field Induced Oxidative Processes in Protein Solutions by Pro- and Antioxidants Sensed by Biochemiluminescence”

Kateřina Červinková[1], Petra Vahalová[1], Michaela Poplová[1], Tomáš Zakar[1], Daniel Havelka[1], Martin Paidar[2], Viliam Kolivoška[3,*], and Michal Cifra[1,]**

[1]Institute of Photonics and Electronics of the Czech Academy of Sciences, Prague, Czechia
[2]Department of Inorganic Technology, Faculty of Chemical Technology, University of Chemistry and Technology, Technická 5, Prague, 160 28, Czechia
[3]J. Heyrovský Institute of Physical Chemistry of the Czech Academy of Sciences, Prague, 18200, Czechia
[*]viliam.kolivoska@jh-inst.cas.cz
[**]cifra@ufe.cz

ABSTRACT

This supplementary information provides extensive additional data and methodological details on BAL measurements that complement the findings in the main manuscript. Included are supplementary figures and tables that enhance the understanding of the experimental results. A critical table details the rate constants of bulk chemical reactions mentioned in the proposed scheme.

Rate constants of bulk chemical reactions

Here, we provide essential details on the rate constants of the bulk chemical reactions discussed in the main text of our study. Accurate determination of these constants is crucial for understanding the kinetics and dynamics of the proposed reaction scheme 2.

Table SI 1-1. Rate constants of bulk chemical reactions

ID	Reaction	Value	Ref.
5A	$2HO^\bullet \rightarrow \frac{1}{4}(^1O_2 + ^3O_2) + H_2O$	5.5×10^9 M^{-1}s^{-1}	1
		5×10^9 M^{-1}s^{-1}	2
5B	$2HO^\bullet \rightarrow H_2O_2$	4.7×10^9 M^{-1}s^{-1}	3
		$(4 \pm 1) \times 10^9$ M^{-1}s^{-1}	4
		5.5×10^9 M^{-1}s^{-1}	5
6	$HO^\bullet + HO_2^\bullet \rightarrow \frac{1}{2}(^1O_2 + ^3O_2) + H_2O$	7.1×10^9 M^{-1}s^{-1}	1
		6×10^9 M^{-1}s^{-1}	4
		6×10^9 M^{-1}s^{-1}	5
7	$2HO_2^\bullet \rightarrow \frac{1}{2}(^1O_2 + ^3O_2) + H_2O_2$	8.3×10^5 M^{-1}s^{-1}	1
		$(3.4 \pm 2.5) \times 10^6$ M^{-1}s^{-1}	4
		$(8.6 \pm 0.62) \times 10^5$ M^{-1}s^{-1}	6
		$(8.3 \pm 0.7) \times 10^5$ M^{-1}s^{-1}	7
8	$^1O_2 \rightarrow ^3O_2 + h\nu$	0.11 s^{-1}	8
		1 s^{-1}	9
		0.1 s^{-1}	10
n/a	$^1O_2 \rightarrow ^3O_2$ (radiationless transition)	2.9×10^5 s^{-1}	11
		2.8×10^5 s^{-1}	12
		4.4×10^5 s^{-1}	13

Continued on next page

Table SI 1-1 – continued from previous page

ID	Reaction	Value	Ref.
9	$^1O_2 + ^1O_2 \rightarrow ^3O_2 + ^3O_2 + h\nu$	$(3.3 \pm 0.6) \times 10^{-2}$ $M^{-1}s^{-1}$	14
		$(2.65 \pm 0.8) \times 10^{-2}$ $M^{-1}s^{-1}$	15
		0.1 $M^{-1}s^{-1}$	1
11	$HO_2^\bullet \rightarrow H^+ + O_2^{\bullet-}$	7.5×10^6 s^{-1}	1
		1.6×10^5 s^{-1}	16
		7.9×10^5 s^{-1}	17
12	$H_2O_2 + O_2^{\bullet-} \rightarrow HO^\bullet + OH^- + ^3O_2$	16 $M^{-1}s^{-1}$	1
		(0.13 ± 0.07) $M^{-1}s^{-1}$	18
		0.005 - 2.25 $M^{-1}s^{-1}$	19
13	$HO^\bullet + H_2O_2 \rightarrow HO_2^\bullet + H_2O$	3×10^7 $M^{-1}s^{-1}$	1
		$(2.6 \pm 0.8) \times 10^7$ $M^{-1}s^{-1}$	4
		2.7×10^7 $M^{-1}s^{-1}$	5
15	$HO^\bullet + BSA \rightarrow H_2O + BSA^\bullet$	reactions of $HO^\bullet$ with amino acids:	
		His: 4.8×10^9 $M^{-1}s^{-1}$	20
		4.3 - 5.0 $\times 10^9$ $M^{-1}s^{-1}$	21
		1.9 - 5.0 $\times 10^9$ $M^{-1}s^{-1}$	4
		Trp: 13×10^9 $M^{-1}s^{-1}$	20
		7.1 - 14 $\times 10^9$ $M^{-1}s^{-1}$	21
		Tyr: 9.4 - 10.5 $\times 10^9$ $M^{-1}s^{-1}$	21
		Phe: 5.8 - 7.2 $\times 10^9$ $M^{-1}s^{-1}$	21
		5.8 - 7.2 $\times 10^9$ $M^{-1}s^{-1}$	4
		Arg: 0.73 - 5.7 $\times 10^9$ $M^{-1}s^{-1}$	21
		0.57 - 3.5 $\times 10^9$ $M^{-1}s^{-1}$	4
		Cys: 19×10^9 $M^{-1}s^{-1}$	20
		5.9 - 40 $\times 10^9$ $M^{-1}s^{-1}$	4
		Met: 7.4×10^9 $M^{-1}s^{-1}$	20
		6.0 - 8.2 $\times 10^9$ $M^{-1}s^{-1}$	21
		6.5 - 8.5 $\times 10^9$ $M^{-1}s^{-1}$	4
		Val: 0.66 - 0.67 $\times 10^9$ $M^{-1}s^{-1}$	21
		0.66 - 0.72 $\times 10^9$ $M^{-1}s^{-1}$	4
		Leu: 1.6 - 1.9 $\times 10^9$ $M^{-1}s^{-1}$	21
		1.6 - 1.8 $\times 10^9$ $M^{-1}s^{-1}$	4
		Ile: 1.7×10^9 $M^{-1}s^{-1}$	21
		1.7 - 1.8 $\times 10^9$ $M^{-1}s^{-1}$	4
		Gly: 0.0073 - 0.017 $\times 10^9$ $M^{-1}s^{-1}$	21
		0.0046 - 0.26 $\times 10^9$ $M^{-1}s^{-1}$	4
		Ala: 0.074 - 0.079 $\times 10^9$ $M^{-1}s^{-1}$	21
		0.046×10^9 $M^{-1}s^{-1}$	4
		Ser: 0.23 - 0.32 $\times 10^9$ $M^{-1}s^{-1}$	21
		0.25 - 0.32 $\times 10^9$ $M^{-1}s^{-1}$	4
		Thr: 0.36 - 0.51 $\times 10^9$ $M^{-1}s^{-1}$	21
		0.39 - 0.51 $\times 10^9$ $M^{-1}s^{-1}$	4
		Lys: 0.35 - 0.60 $\times 10^9$ $M^{-1}s^{-1}$	21
		Lys: 0.35 - 0.65 $\times 10^9$ $M^{-1}s^{-1}$	4
		Pro: 0.28 - 0.65 $\times 10^9$ $M^{-1}s^{-1}$	21
		0.31 - 0.65 $\times 10^9$ $M^{-1}s^{-1}$	4
		Asp: 0.031 - 0.075 $\times 10^9$ $M^{-1}s^{-1}$	21
		0.033 - 0.075 $\times 10^9$ $M^{-1}s^{-1}$	4
		Asn: 0.049×10^9 $M^{-1}s^{-1}$	21
		0.032 - 0.049 $\times 10^9$ $M^{-1}s^{-1}$	4
		Glu: 0.127 - 0.23 $\times 10^9$ $M^{-1}s^{-1}$	21

Continued on next page

Table SI 1-1 – continued from previous page

ID	Reaction	Value	Ref.
		0.14 - 0.23 $\times$ 10^9 $M^{-1}s^{-1}$	4
		Gln: 0.54 $\times$ 10^9 $M^{-1}s^{-1}$	21
		0.16 - 0.54 $\times$ 10^9 $M^{-1}s^{-1}$	4
n/a	$BSA + ^1O_2 \rightarrow BSAOOH$	reactions of aromatic aminoacids with $^1O_2$	
		His: 3.4 $\times$ 10^7 $M^{-1}s^{-1}$	22
		Trp: 1.3 $\times$ 10^7 $M^{-1}s^{-1}$	22
		Tyr: 0.2 - 0.5 $\times$ 10^7 $M^{-1}s^{-1}$	22
		Phe: 0.07 $\times$ 10^7 $M^{-1}s^{-1}$	22
n/a	$BSA + O_3 \rightarrow$ product	reactions of aromatic aminoacids with O_3	
		His: 1.7 $\times$ 10^3 $M^{-1}s^{-1}$	23
		Trp: 5.4 $\times$ 10^5 $M^{-1}s^{-1}$	23
		Phe: 2.1 $\times$ 10^3 $M^{-1}s^{-1}$	23
16	$BSA^{\bullet} + ^3O_2 \rightarrow BSAOO^{\bullet}$	reactions of organic radicals $R^{\bullet}$ with $^3O_2$ to produce $ROO^{\bullet}$ have rate constants in the range:	
		10^8 - 10^{10} $M^{-1}s^{-1}$	24
17	$2BSAOO^{\bullet} \rightarrow\rightarrow ^3O_2 + ^3(R'COR'')^* + ^1O_2$	peroxyradicals react with organic matter with rate constants in the range:	
		10^2 to 10^8 $M^{-1}s^{-1}$	24
18	$^3(R'COR'')^* \rightarrow ^1(R'COR'') + h\nu$	R' = R'' = CH_3 (acetone): $4.6 \times 10^4 s^{-1}$	25
19	$H_2O_2 + Fe^{2+} \rightarrow HO^{\bullet} + OH^- + Fe^{3+}$	56 $M^{-1}s^{-1}$	1
		76 $M^{-1}s^{-1}$	26
		63 $M^{-1}s^{-1}$	16
20	$H_2O_2 + Fe^{3+} \rightarrow HO_2^{\bullet} + H^+ + Fe^{2+}$	0.01 $M^{-1}s^{-1}$	16
		0.01 - 0.02 $M^{-1}s^{-1}$	17

Supplementary figures on luminescence measurements and current transients

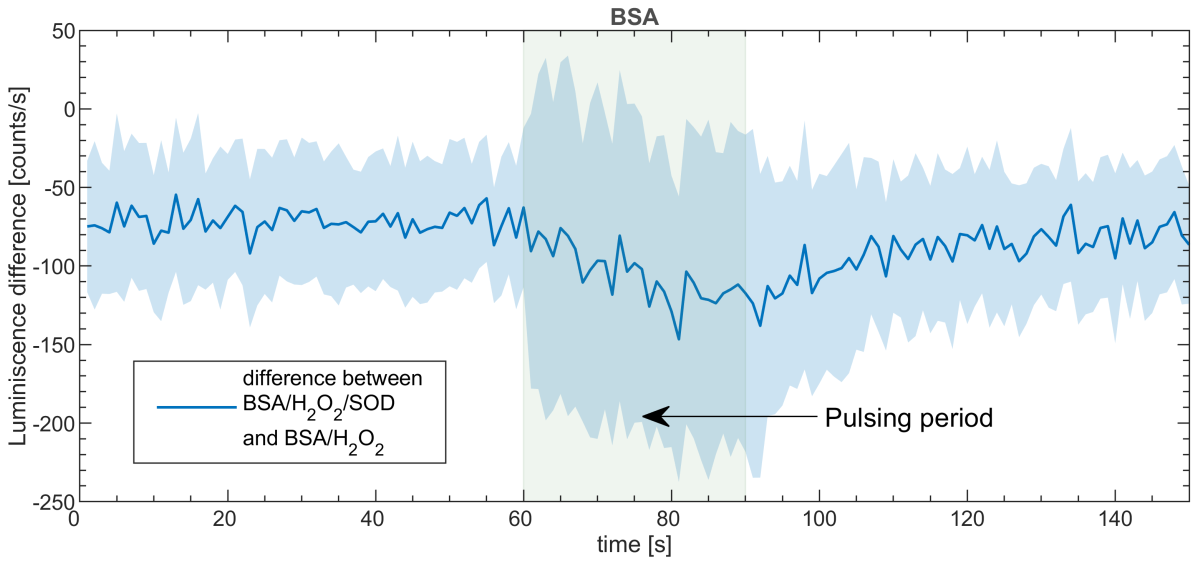

Figure SI 1-1. Mathematical difference of averaged luminescence transients obtained in PEF experiments performed with BSA/H_2O_2/SOD and BSA/H_2O_2.

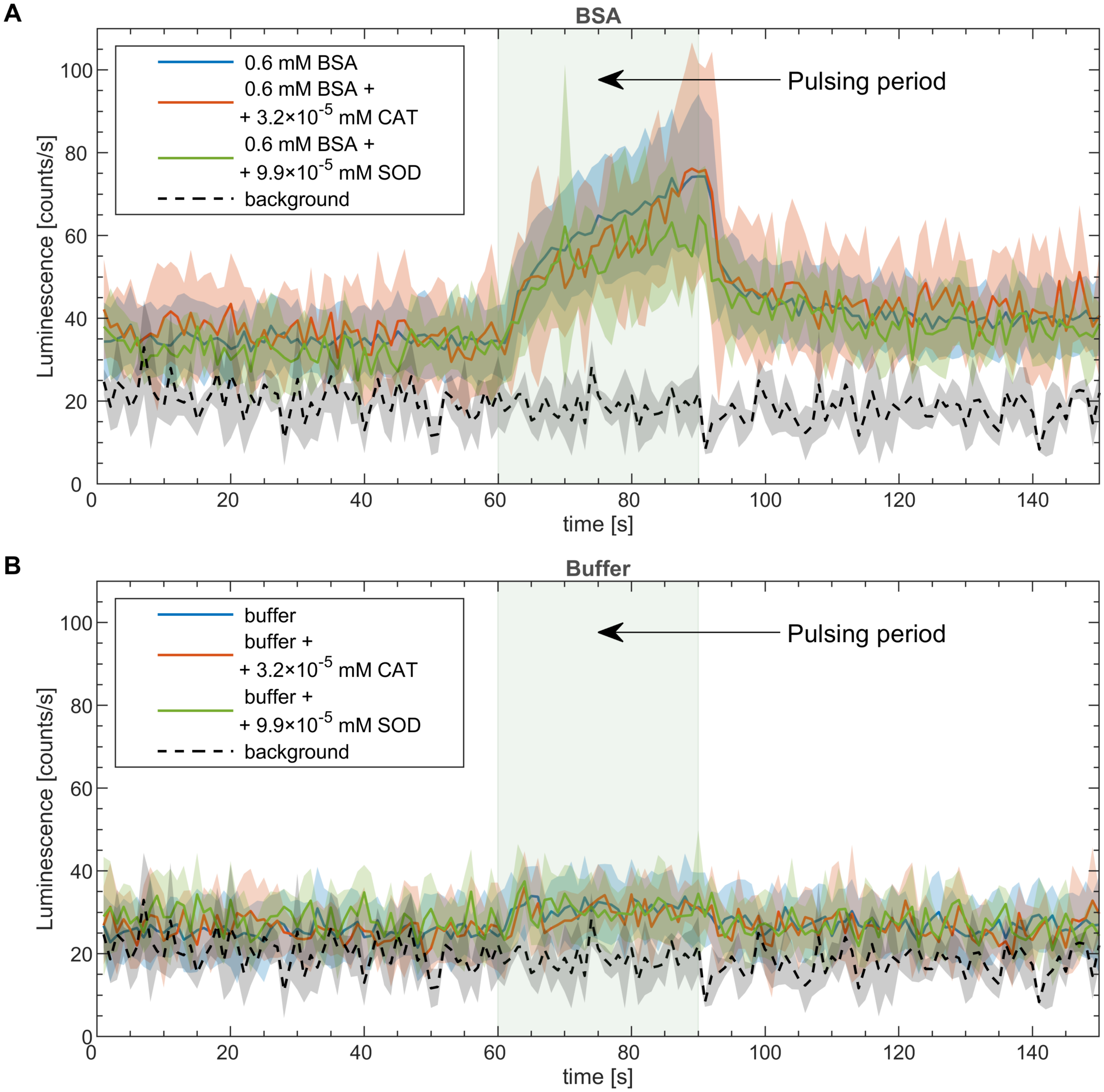

Figure SI 1-2. Averaged luminescence transients obtained in PEF experiments performed with (A) BSA and (B) PB in the absence of H_2O_2 and with additionally introduced antioxidant enzymes (CAT and SOD).

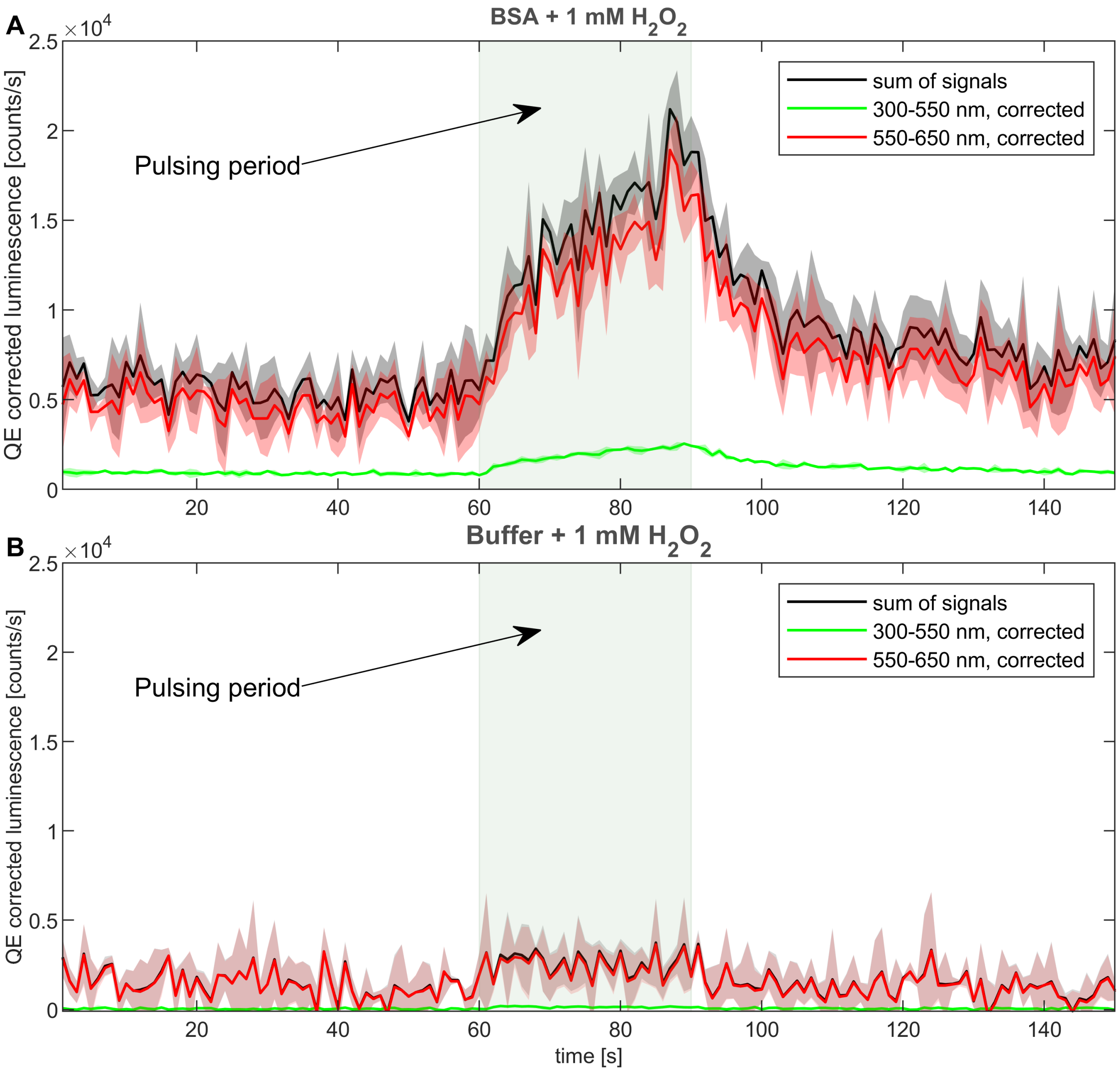

Figure SI 1-3. Averaged luminescence transients obtained in PEF experiments performed with (A) BSA and (B) PB solution, both additionally containing 1 mM H_2O_2, with luminescence being measured separately in the wavelength range of 300 nm to 550 nm and 550 nm to 650 nm. Shown data are corrected for the averaged QE of the employed PMT in the respective wavelength range. The mathematical sum of corrected signals obtained in both wavelength ranges is additionally plotted.

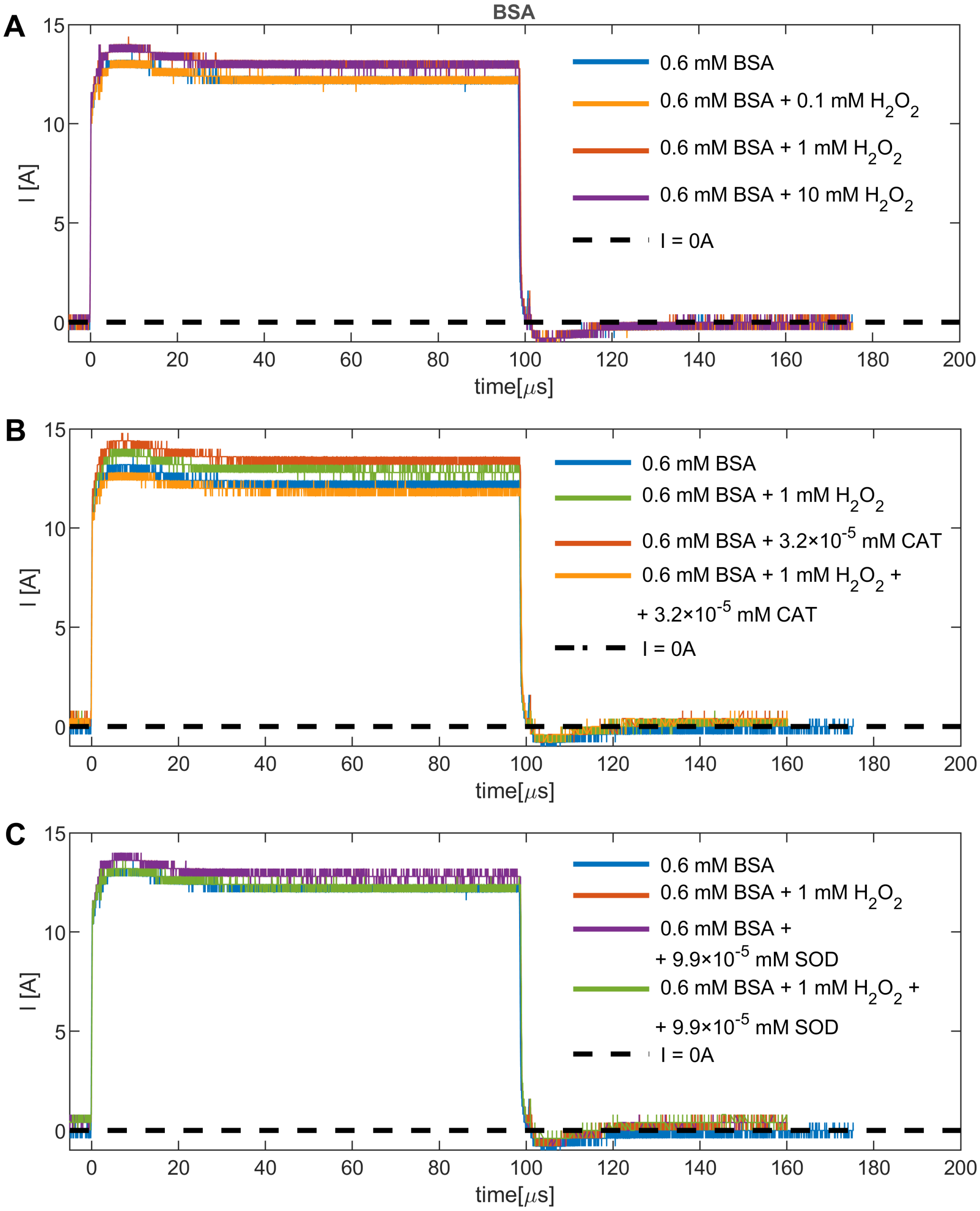

Figure SI 1-4. Representative current transients obtained in the first pulse within the PEF treatment for (A) BSA solution with varied concentration of H_2O_2, (B) BSA solution in the absence and presence of CAT and with additionally introduced H_2O_2, (C) BSA solution in the absence and presence of SOD and with additionally introduced H_2O_2.

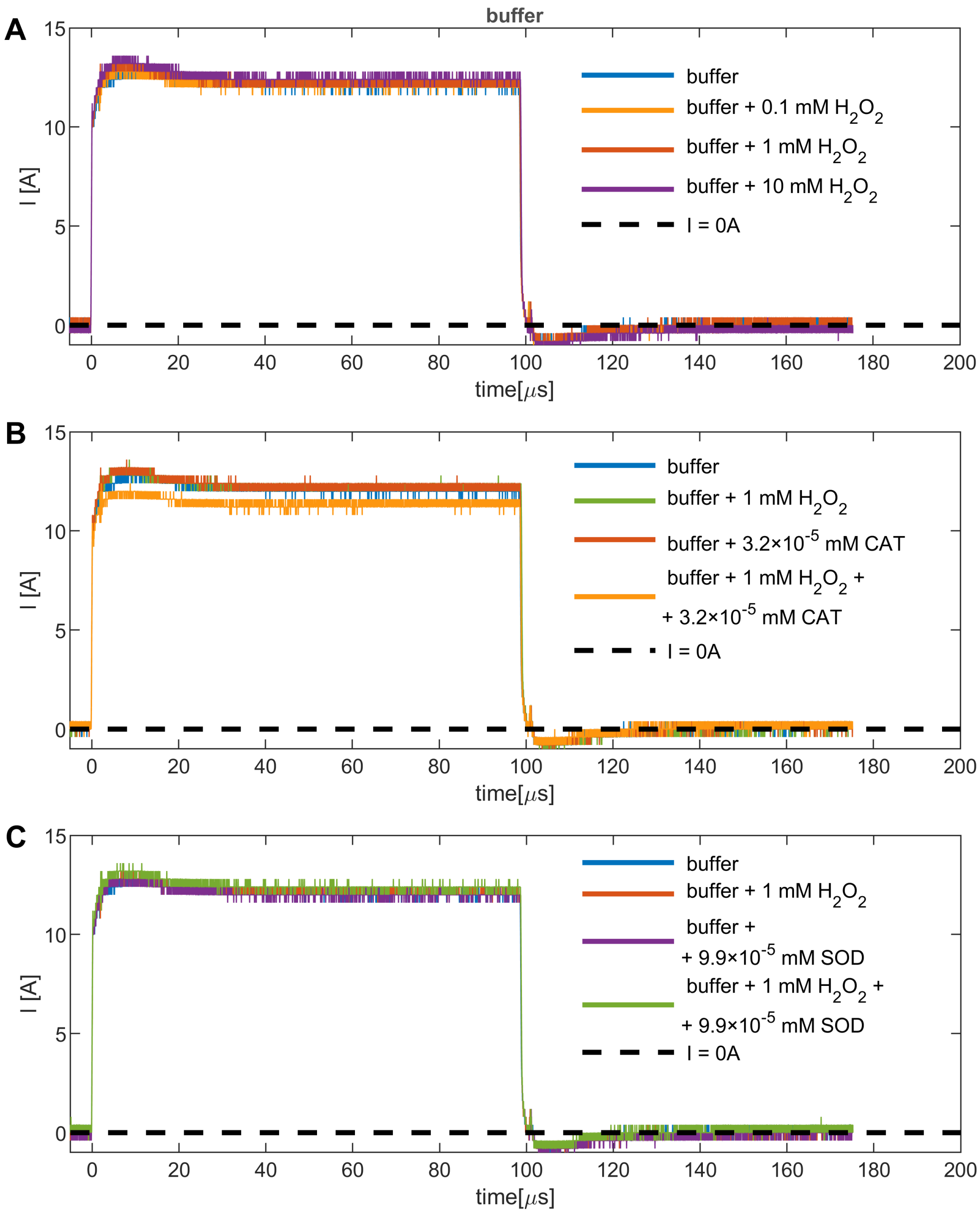

Figure SI 1-5. Representative current transients obtained in the first pulse within the PEF treatment for (A) PB with varied concentration of H_2O_2, (B) PB in the absence and presence of CAT and with additionally introduced H_2O_2, (C) PB in the absence and presence of SOD and with additionally introduced H_2O_2. Results demonstrate that these additions have negligible influence on observed currents and so also on sample conductivity.

Suplementry tables on luminescence and fluorescence measurements

Table SI 1-2. Mean values and standard deviations of luminescence integrals obtained in the pre-pulsing, pulsing and post-pulsing period (common time basis of 30 s) for BSA and PB with varied concentration of H_2O_2. Mean values of luminescence are shown as graphs in Fig. 3E, G.

BSA: Luminescence [counts]								
$c(H_2O_2)$[mM]	0		0.1		1		10	
	Mean	STD	Mean	STD	Mean	STD	Mean	STD
pre-pulsing	1039	140	2370	797	9128	2750	29145	1677
pulsing	1864	315	5476	2898	182359	5641	54229	987
post-pulsing	1336	163	5138	2511	13372	4097	41840	1549
**buffer: Luminescence [counts]**								
$c(H_2O_2)$[mM]	0		0.1		1		10	
	Mean	STD	Mean	STD	Mean	STD	Mean	STD
pre-pulsing	774	137	718	51	916	144	1170	222
pulsing	937	112	1007	42	1399	199	2035	71
post-pulsing	817	147	788	20	971	112	1307	77

Table SI 1-3. Fluorescence intensity [a.u.] of ANS obtained at emission maximum (481 nm) for PEF treated, control and sham BSA and BSA/H_2O_2 samples. Mean and STD for the data shown in Fig.5G,H

sample	BSA		BSA + 1 mM H_2O_2	
	Mean	STD	Mean	STD
control	17787	1943	17316	1693
sham	17844	1624	18023	2135
PEF	18945	941	19358	1335

Table SI 1-4. Number (N) of experimental repetitions used in the data presented in this paper to calculate mean and STD values

Sample	N	Figure
background	3	3A, 3B, 3D, 3F, 4A, 4B, SI 1-2A, SI 1-2B
BSA	33	3A, 3B, 3F, 4A, SI 1-2A
BSA/0.1 mM H_2O_2	6	3F
BSA/1 mM H_2O_2	33	3A, 3B, 3F, 4A
BSA/10 mM H_2O_2	6	3F
BSA/CAT	6	SI 1-2A
BSA/SOD	6	SI 1-2A
BSA/1 mM H_2O_2/CAT	6	4A
BSA/1 mM H_2O_2/SOD	6	4A
BSA/1 mM H_2O_2/optical filters	3	SI 1-3A
PB	17	3A, 3B, 3D, SI 1-2B
PB/0.1 mM H_2O_2	5	3D
PB/1 mM H_2O_2	12	3A, 3B, 3D, 4B
PB/10 mM H_2O_2	6	3D
PB/CAT	6	SI 1-2B
PB/SOD	6	SI 1-2B

Continued on next page

Table SI 1-4 – continued from previous page

Sample	N	Figure
PB/1 mM H_2O_2/CAT	6	4B
PB/1 mM H_2O_2/SOD	6	4B
PB/1 mM H_2O_2/optical filters	3	SI 1-3B
temperature	3	11
sulfhydryl groups	3	5D
carbonyl groups	4	5C
tryptophan and tyrosine fluorescence	3	5E, 5F
ANS fluorescence	9	5G, 5H
dynamic light scattering	3	5A, 5B

Amino acid composition of BSA

Residue Type	Count	Frequency in BSA (%)	Average Frequency across Proteome (%)[27]	Relative Difference (BSA-AVG)/AVG
Ala	48	7.9	8.7	9.2
Arg	26	4.3	6.2	-30.6
Asn	14	2.3	3.9	-41.0
Asp	59	9.7	5.1	90.2
Cys	40	6.6	1.5	340.0
Gln	20	3.3	3.9	15.4
Glu	30	4.9	6.2	-21.0
Gly	15	2.5	6.8	-63.2
His	30	4.9	2.2	122.7
Ile	60	9.9	5.7	73.7
Leu	55	9.1	9.8	7.1
Lys	65	10.7	5.3	101.9
Met	5	0.8	2.4	-66.7
Phe	17	2.8	4.0	-30.0
Pro	28	4.6	5.0	8.0
Ser	32	5.3	7.0	-24.3
Thr	34	5.6	5.3	5.7
Trp	3	0.5	1.3	-61.5
Tyr	21	3.5	2.9	20.7
Val	38	6.3	6.5	3.1

Table SI 1-5. Amino acid composition of BSA, obtained using https://proteininformationresource.org/pirwww/search/comp_mw.shtml from a sequence from UniProt protein database code P02769, https://www.uniprot.org/uniprotkb/P02769/entry. The average value is from many proteins[27], see http://proteopedia.org/w/Amino_acid_composition. The value is highlighted in blue when it exceeds 20% of relative difference of the average value and in yellow if it's lower than 20% the average value.

Raw data description

The source data can be found underhttps://doi.org/10.5281/zenodo.12542774 and are sorted according to the figure number. For plotting the mean and the standard deviation of several independent measurements in the Fig. 3, 4, 5, SI 1-3, SI 1-2, SI 1-1, and SI 1-3, we used a function 'stdshade' [Musall, S. (2024). stdshade. MATLAB Central File Exchange. Retrieved June 26, 2024, from https://www.mathworks.com/matlabcentral/fileexchange/29534-stdshade]. Fig. 9 was ploted by the predefined Matlab function 'shadedErrorBar'.

References

1. Ivanova, I. P. *et al.* Mechanism of chemiluminescence in Fenton reaction. *Journal of Biophysical Chemistry* **03**, 88–100 (2012). URL http://www.scirp.org/journal/doi.aspx?DOI=10.4236/jbpc.2012.31011.

2. Collin, F. Chemical Basis of Reactive Oxygen Species Reactivity and Involvement in Neurodegenerative Diseases. *International Journal of Molecular Sciences* **20**, 2407 (2019). URL https://www.mdpi.com/1422-0067/20/10/2407. Number: 10 Publisher: Multidisciplinary Digital Publishing Institute.

3. Crowell, R. A., Lian, R., Sauer, M. C., Oulianov, D. A. & Shkrob, I. A. Geminate recombination of hydroxyl radicals generated in 200 nm photodissociation of aqueous hydrogen peroxide. *Chemical Physics Letters* **383**, 481–485 (2004). URL https://linkinghub.elsevier.com/retrieve/pii/S000926140302044X.

4. Ross, F. & Ross, A. B. Selected specific rates of reactions of transients from water in aqueous solution. III. Hydroxyl radical and perhydroxyl radical and their radical ions. Tech. Rep. NSRDS-NBS-59, Notre Dame Univ., IN (USA). Radiation Lab. (1977). URL https://www.osti.gov/biblio/6635906.

5. Buxton, G. V., Greenstock, C. L., Helman, W. P. & Ross, A. B. Critical Review of rate constants for reactions of hydrated electrons, hydrogen atoms and hydroxyl radicals (.OH/.O $^-$ in Aqueous Solution. *Journal of Physical and Chemical Reference Data* **17**, 513–886 (1988). URL http://aip.scitation.org/doi/10.1063/1.555805.

6. Bielski, B. H. J. Reevaluation of the spectral and kinetic properties of HO_2 and O_2^- free radicals. *Photochemistry and Photobiology* **28**, 645–649 (1978). URL https://onlinelibrary.wiley.com/doi/10.1111/j.1751-1097.1978.tb06986.x.

7. Bielski, B. H. J., Cabelli, D. E., Arudi, R. L. & Ross, A. B. Reactivity of HO_2/O^-_2 Radicals in Aqueous Solution. *Journal of Physical and Chemical Reference Data* **14**, 1041–1100 (1985). URL http://aip.scitation.org/doi/10.1063/1.555739.

8. Poulsen, T. D., Ogilby, P. R. & Mikkelsen, K. V. Solvent Effects on the $O_2(a\Delta_g)$-$O_2(X^3\sigma_g^-)$ Radiative Transition: Comments Regarding Charge-Transfer Interactions. *The Journal of Physical Chemistry A* **102**, 9829–9832 (1998). URL https://pubs.acs.org/doi/10.1021/jp982567w.

9. Jensen, R. L., Holmegaard, L. & Ogilby, P. R. Temperature Effect on Radiative Lifetimes: The Case of Singlet Oxygen in Liquid Solvents. *The Journal of Physical Chemistry B* **117**, 16227–16235 (2013). URL https://pubs.acs.org/doi/10.1021/jp410185n.

10. Bilski, P., Holt, R. N. & Chignell, C. F. Properties of singlet molecular oxygen O2(1Dg) in binary solvent mixtures of different polarity and proticity. *Journal of Photochemistry and Photobiology A: Chemistry* **109**, 243–249 (1997).

11. Bregnhøj, M., Westberg, M., Jensen, F. & Ogilby, P. R. Solvent-dependent singlet oxygen lifetimes: temperature effects implicate tunneling and charge-transfer interactions. *Physical Chemistry Chemical Physics* **18**, 22946–22961 (2016). URL https://pubs.rsc.org/en/content/articlelanding/2016/cp/c6cp01635a. Publisher: The Royal Society of Chemistry.

12. Davis, C. A., McNeill, K. & Janssen, E. M.-L. Non-Singlet Oxygen Kinetic Solvent Isotope Effects in Aquatic Photochemistry. *Environmental Science & Technology* **52**, 9908–9916 (2018). URL https://doi.org/10.1021/acs.est.8b01512. Publisher: American Chemical Society.

13. Wilkinson, F. & Brummer, J. G. Rate constants for the decay and reactions of the lowest electronically excited singlet state of molecular oxygen in solution. *Journal of Physical and Chemical Reference Data* **10**, 809–999 (1981). URL http://aip.scitation.org/doi/10.1063/1.555655.

14. Borrell, P. & Rich, N. H. The rate constant for the “dimol” transition of singlet oxygen, O 2 (a 1 δ g), and the likely symmetry of the emitting species **99**, 144–147 (1983).

15. Fisk, G. A. & Hays, G. N. A study of the 0.634 pm dimol emission from excited molecular oxygen. *Chemical Physics Letters* **79**, 331–333 (1981).

16. Pontes, R. F., Moraes, J. E., Machulek, A. & Pinto, J. M. A mechanistic kinetic model for phenol degradation by the Fenton process. *Journal of Hazardous Materials* **176**, 402–413 (2010). URL https://linkinghub.elsevier.com/retrieve/pii/S0304389409018287.

17. Burbano, A. A., Dionysiou, D. D. & Suidan, M. T. Effect of oxidant-to-substrate ratios on the degradation of MTBE with Fenton reagent. *Water Research* **42**, 3225–3239 (2008). URL https://linkinghub.elsevier.com/retrieve/pii/S0043135408001413.

18. Weinstein, J. & Bielski, B. H. Kinetics of the interaction of perhydroxyl and superoxide radicals with hydrogen peroxide. The Haber-Weiss reaction **101**, 58–62 (1979).

19. Koppenol, W. The Haber-Weiss cycle – 70 years later. *Redox Report* **6**, 229–234 (2001). URL http://www.tandfonline.com/doi/full/10.1179/135100001101536373.

20. Choe, E. & Min, D. B. Chemistry and Reactions of Reactive Oxygen Species in Foods. *Journal of Food Science* **70**, R142–R159 (2005). URL https://onlinelibrary.wiley.com/doi/10.1111/j.1365-2621.2005.tb08329.x.

21. Masuda, T., Nakano, S. & Kondo, M. Rate constants for the reactions of OH radicals with the enzyme proteins as determined by the p-nitrosodimethylaniline method. *Journal of radiation research* **14**, 339–345 (1973). Publisher: Journal of Radiation Research Editorial Committee.

22. Michaeli, A. & Feitelson, J. REACTIVITY OF SINGLET OXYGEN TOWARD AMINO ACIDS AND PEPTIDES. *Photochemistry and Photobiology* **59**, 284–289 (1994). URL https://onlinelibrary.wiley.com/doi/10.1111/j.1751-1097.1994.tb05035.x.

23. Sharma, V. K. & Graham, N. J. Oxidation of Amino Acids, Peptides and Proteins by Ozone: A Review. *Ozone: Science & Engineering* **32**, 81–90 (2010). URL https://doi.org/10.1080/01919510903510507. Publisher: Taylor & Francis _eprint: https://doi.org/10.1080/01919510903510507.

24. Neta, P., Huie, R. E. & Ross, A. B. Rate Constants for Reactions of Peroxyl Radicals in Fluid Solutions. *Journal of Physical and Chemical Reference Data* **19**, 413–513 (1990). URL https://pubs.aip.org/jpr/article/19/2/413/241425/Rate-Constants-for-Reactions-of-Peroxyl-Radicals.

25. Porter, G., Yip, R. W., Dunston, J. M., Cessna, A. J. & Sugamori, S. E. Detection and lifetime of the triplet state of acetone in solution. *Transactions of the Faraday Society* **67**, 3149–3154 (1971). URL http://pubs.rsc.org/-/content/articlehtml/1971/tf/tf9716703149.

26. Walling, C. Fenton's reagent revisited. *Accounts of Chemical Research* **8**, 125–131 (1975). URL https://pubs.acs.org/doi/abs/10.1021/ar50088a003.

27. Carugo, O. Amino acid composition and protein dimension. *Protein Science* **17**, 2187–2191 (2008). URL https://onlinelibrary.wiley.com/doi/10.1110/ps.037762.108.
